# ChIP-Enrich: gene set enrichment testing for ChIP-seq data

**DOI:** 10.1093/nar/gku463

**Published:** 2014-05-30

**Authors:** Ryan P. Welch, Chee Lee, Paul M. Imbriano, Snehal Patil, Terry E. Weymouth, R. Alex Smith, Laura J. Scott, Maureen A. Sartor

**Affiliations:** 1 Department of Computational Medicine and Bioinformatics, University of Michigan, Ann Arbor, MI 48109, USA; 2 Biostatistics Department, University of Michigan, Ann Arbor, MI 48109, USA; 3 Center for Computational Medicine and Bioinformatics, University of Michigan, Ann Arbor, MI 48109, USA

## Abstract

Gene set enrichment testing can enhance the biological interpretation of ChIP-seq data. Here, we develop a method, ChIP-Enrich, for this analysis which empirically adjusts for gene locus length (the length of the gene body and its surrounding non-coding sequence). Adjustment for gene locus length is necessary because it is often positively associated with the presence of one or more peaks and because many biologically defined gene sets have an excess of genes with longer or shorter gene locus lengths. Unlike alternative methods, ChIP-Enrich can account for the wide range of gene locus length-to-peak presence relationships (observed in ENCODE ChIP-seq data sets). We show that ChIP-Enrich has a well-calibrated type I error rate using permuted ENCODE ChIP-seq data sets; in contrast, two commonly used gene set enrichment methods, Fisher's exact test and the binomial test implemented in Genomic Regions Enrichment of Annotations Tool (GREAT), can have highly inflated type I error rates and biases in ranking. We identify DNA-binding proteins, including CTCF, JunD and glucocorticoid receptor α (GRα), that show different enrichment patterns for peaks closer to versus further from transcription start sites. We also identify known and potential new biological functions of GRα. ChIP-Enrich is available as a web interface (http://chip-enrich.med.umich.edu) and Bioconductor package.

## INTRODUCTION

Genome-wide high-throughput experiments can assess transcription factor binding, epigenetic marks, differential gene expression or disease association, and often result in thousands of identified genomic regions or genes. Gene set enrichment testing is one way to determine how these lists of genomic regions or genes are related biologically, e.g. by assessment of Gene Ontology (GO) terms (1–3). For ChIP-seq experiments, oftentimes thousands of transcription factor binding sites or histone modification sites are identified. Enrichment testing of this data, or with a union or intersection of multiple ChIP-seq data sets, can identify key biological processes, functions, disease gene signatures or other biological concepts regulated by the factor(s) under the given experimental conditions (4). Conversely, ChIP-seq data can be used to create gene sets against which other experimental data sets can be tested for significant enrichment, including other ChIP-seq data (5,6).

Gene set enrichment tests can generally be classified as competitive (2,7,8), self-contained (9) or a hybrid (9,10), as discussed by Efron and Tibshirani in (11). The hypothesis of competitive approaches is that there is a higher proportion of identified genes (or a higher level of significance overall) in the gene set of interest than in the remaining genes. In contrast, self-contained methods only use information about the genes in the gene set of interest, and test whether the significance level of the set is greater than expected given a null hypothesis. The enrichment testing methods used for sets of genomic regions (ChIP-seq data), including Fisher's exact test (FET) and binomial based tests, are all competitive approaches (3,12).

FET, and slight variations on it, has traditionally been used for gene set enrichment in microarray gene expression data (2,13–16). FET makes the assumption that each gene has an equal probability of being identified as significant. Across gene sets, this means that each gene set is expected under the null hypothesis to have approximately the same proportion of significant genes as the overall proportion of significant genes. In contrast to microarray data, the data generated from ChIP-seq, RNA-seq and genome-wide association studies (GWASs) often show a positive correlation between the length of the relevant genomic region and detection of the gene (17–19). In ChIP-seq data, the probability of a peak occurring within a gene or its surrounding non-coding sequence, which together we denote as the gene locus, is often positively correlated with the length of the locus (20). Due to this correlation, genes with longer locus lengths contribute a disproportionate amount to the enrichment signal, and this bias introduced in the signal due to gene locus length violates the assumptions of FET. Furthermore, because many commonly tested gene sets contain genes with substantially longer (e.g. developmentally and nervous system-related genes) or shorter (e.g. electron transport, ribosomal ribonucleic acid (rRNA) processing) than average locus length (20), the gene sets with longer or shorter than average locus length are more or less likely, respectively, to be detected as significantly enriched (18). Therefore, lack of effective adjustment for gene locus length can lead to false positive findings.

Several approaches have been developed to adjust for locus length in ChIP-seq (12), RNA-seq (for example, GOseq (19)) and GWAS data (17,21). For ChIP-seq data, a commonly used binomial-based test asks if the total number of peaks within the loci in a gene set is greater than expected, given the total locus length of the gene set, the total number of peaks and the corresponding length of the genome (implemented in Genomic Regions Enrichment of Annotations Tool (GREAT)) (12,18). In contrast to FET, the assumptions of the binomial test are met when the number of peaks in a locus is proportional to locus length and the variability of peak counts among genes, given gene locus length, is consistent with that expected by the binomial distribution.

We examined the gene locus length-to-peak presence relationships in 63 ENCODE ChIP-seq GM12878 data sets and found they ranged from no relationship to strongly positively correlated. Given these observations, our goal was to develop a gene set enrichment method for ChIP-seq data (ChIP-Enrich) that empirically models and adjusts for the relationship between locus length and peak presence. ChIP-Enrich maintained the expected type I error rate (false positive rate) in all tested data sets, whereas FET and the binomial test did not. For each deoxyribonucleic acid (DNA)-binding protein (DBP), we asked if different (potential) regulatory region definitions would identify different enriched/disenriched gene sets. For the glucocorticoid receptor α (GRα), we examined the ability of ChIP-Enrich to detect known and potentially novel functions. Our method is freely available in the R Bioconductor package *chipenrich* and as a web-based program (http://chip-enrich.med.umich.edu).

## MATERIALS AND METHODS

### Experimental ChIP-seq peak data sets

We used ENCODE ChIP-seq peak data sets from 63 DBPs for cell line GM12878 (22) (see http://chip-enrich.med.umich.edu/summaryReport.jsp) (Supplementary Table S1). We used the existing peak calls, which were called by the original authors using one or two of three peak calling methods (MACS, spp or Scripture (23–25)). For the subset of data sets that were called by two callers (MACS and spp), we use results from MACS, as we generally observed a larger number of called peaks for MACS than for spp.

### Gene loci definitions and presence of peaks in a locus

We define a gene as the region between the furthest upstream transcription start site (TSS) and furthest downstream transcription end site (TES) for that gene. The positions of the TSSs and TESs for each gene were extracted from the UCSC knownGene table (human genome build hg19). We removed small nuclear RNAs as they are likely to have different regulatory mechanisms than other genes and often reside within the boundaries of other genes. For gene set enrichment testing we assign ChIP-seq peaks to genes (based on the peak midpoint) using three primary definitions of a gene's designated regulatory region (locus definitions). (i) ‘Nearest TSS’: the region between the upstream and downstream midpoints between a gene and the two adjacent genes’ TSSs. This is equivalent to assigning each peak to the gene with the nearest TSS. (ii) ‘Nearest gene’: the region from the midpoint between the TSS and the adjacent gene's TSS or TSE (whichever is closest) to the midpoint between the TES and the adjacent gene's TSS or TES (whichever is closest). This is equivalent to assigning peaks to the nearest gene. (iii) ‘≤1 kb from TSS’: the region within 1 kb of all TSSs in a gene. If TSSs from the adjacent gene(s) are less than 2 kb away, we use the midpoint between the two TSSs as the boundary of the locus for each gene. Additionally we define ‘≤5 kb from TSS’, using the same rules as we defined ‘≤1 kb from TSS’, and we define ‘>10 kb from TSS’, by subtracting the 10-kb regions around the TSS from the ‘nearest TSS’ locus definition. We define peak presence in a locus as ≥1 peak midpoint within the gene locus boundaries.

### GO terms

GO terms from GO molecular functions, GO cellular components and GO biological processes were extracted from Bioconductor species specific annotation packages and the *GO.db* R package. We removed genes from each GO term that do not exist in our gene locus definitions as these genes could not have a peak assigned to them. For testing in the manuscript and in our tool, we exclude GO terms with <10 genes as they have more limited power to detect significant results, and as a rule of thumb logistic regression requires at least 10 events for each explaining variable (26). In the manuscript, we also exclude reporting GO terms with >500 genes, as the categories become broader and less informative in interpreting the results. *Q*-values were calculated using all GO terms with 10–2000 genes (our tool's defaults).

### Overdispersion test of peak count (given locus length) in each gene set

Overdispersion is defined as higher variability in a data set than expected based on the distribution used to model it. The binomial test in GREAT uses a binomial distribution to model the combined number of peaks for all genes in a gene set, so if significant overdispersion in peak counts exists among genes, the binomial distribution assumption is not satisfied. We tested for overdispersion in the number of peaks per gene (given locus length) in each gene set using Tarone's Z statistic (27). Tarone's Z allows better estimates of overdispersion when the binomial probabilities are close to 0 or 1 (the probabilities of having a peak for each base pair are very close to 0). We tested all gene sets with a minimum of 50 genes (as gene sets with fewer genes often do not have adequate power for this test) and a maximum of 500 genes (the maximum gene set size used throughout the paper). For each DBP, we reported the proportion of gene sets that had significantly higher variability than expected based on the binomial distribution (*q*-value ≤0.05).

### Mappability calculations

To estimate the mappable proportion of each gene locus for different read lengths, we first calculated base pair mappability for reads of lengths 24, 36, 40, 50, 75 and 100 base pairs using mappability data for *Homo sapiens* (build hg19) from the UCSC Genome Browser. The UCSC browser mappability tracks provide, for each base pair *i*, the reciprocal of the number of locations in the genome to which a read beginning at *i* and extending for read length *K* could map; a value of 1 indicates the read maps to one location in the genome, a smaller value indicates the read maps to two or more locations. We set reads with mappability <1 to 0 and calculated base pair mappability as the average read mappability of all possible reads of size *K* that include a specific base pair location, *i*:

(1) \begin{equation*}
B_i = \left( {\frac{1}{{2K - 1}}} \right)\sum\limits_{j = i - K + 1}^{i + (K - 1)} {M_j },
\end{equation*}

where *B_i_* is the mappability of base pair *i* and *M_j_* is the read mappability (based on UCSC's mappability track) of a read of length *K* beginning at position *j*. We define gene locus mappability, *m*, as the average of all base pair mappability, *B_i_*, values for a gene locus; each gene locus mappability score *m* represents the proportion of the gene locus that is uniquely mappable (given the read length of the data).

### ChIP-Enrich method

We developed a logistic regression approach to test for gene set enrichment while adjusting for log_10_ mappable locus length for each gene. Suppose that for a given set of genomic regions (referred to as peaks), we have assigned each peak to a gene locus. The dependent variable is a binary vector defined as 1 if ≥1 peak is assigned to a gene's locus, and 0 if none are assigned to the gene's locus. For each gene set, the explanatory variable of interest is gene set membership, *g*, defined as 1 for genes in the gene set, and 0 for all other genes. Let *L* be the locus length, such that *m·L* is the mappable locus length. Let *π* be the probability that a gene with gene set membership *g*, and adjusted for mappable locus length, has ≥1 peak. Then ${\pi /(1- \pi)}$ are the corresponding odds that a gene, given *g =* 0 or 1 and mappable locus length *m·L*, has ≥1 peak. If the log-odds differ by gene set membership adjusted for (mappable) locus length, then we conclude that peak presence is associated with the gene set. Our model is

(2) \begin{equation*}
\log \frac{\pi }{{1 - \pi }} = \beta _0 + \beta _1 g + f\left( {\log _{10} \left( {mL + 1} \right)} \right),
\end{equation*}

where *β_0_* is the intercept, *β_1_* is the coefficient of interest and the function *f*(log_10_ (*m·L*+1)) is a binomial cubic smoothing spline term that adjusts for log_10_ mappable locus length (or log_10_ locus length if *m* is omitted). We apply the log_10_ transformation to locus length as this improves the model fit (data not shown). The smoothing spline is estimated with a penalized spline using a cubic spline basis fit with 10 knots distributed evenly throughout the data. Placing a knot at each data point as in a true smoothing spline would not be computationally feasible. The model is fit using penalized likelihood maximization, where the smoothing penalty is the squared second derivative penalty, and generalized cross-validation is used to choose the optimal value for the smoothing parameter, *λ* (28,29). We use the *gam* function of the R package *mgcv* to fit the model (30) and the Wald statistic to test for significance of the gene set term, *β*_1_, which is calculated as

(3) \begin{equation*}
W = \left( {\frac{{\hat \beta _1 }}{{s_{\hat \beta 1} }}} \right)^2 ,
\end{equation*}

where $\hat \beta _1$ is the penalized maximum likelihood estimate for *β_1_* and $s_{{\rm \hat \beta 1}}$ is the standard error for $\hat \beta _1$. *W* is distributed as *χ^2^* with one degree of freedom under the null hypothesis *β*_1_ = 0, and *P*-values are calculated accordingly for the alternative hypothesis, *β*_1_ ≠ 0. *P*-values for the gene sets are corrected for multiple testing using the Benjamini–Hochberg false discovery rate approach (31). To be included in the analysis, genes had to be annotated in GO and have a locus defined. For example, we have 19 051 human genes with the ‘nearest TSS’ locus definition and 16 653 (87.4%) of these genes have ≥1 GO term annotation (with no restriction for GO term size).

### R package and website

Our ChIP-Enrich gene set enrichment testing method is implemented in the *chipenrich* package for the R statistical software environment and available through Bioconductor, and as a web version at http://chip-enrich.med.umich.edu/. We also provide FET as an alternative enrichment method. In addition to GO, we include 12 additional annotation sources containing over 20 000 total gene sets (32). We currently support the human genome (hg19), mouse genome (mm9, mm10) and rat genome (rn4). Precomputed mappability is available for hg19 (for read lengths specified above) and for mm9 (read lengths 36, 40, 50, 75 and 100 base pairs). Users may either supply an R data frame (for the R package) or a BED format file containing the peak locations as input. Runtime is typically 10–14 min for testing all GO terms but varies depending on the data set, number of cores and choice of locus definition. In addition to the ‘nearest TSS’, ‘nearest gene’, ‘≤1 kb from TSS’ and ‘≤5 kb from TSS’ locus definitions, described above, in ChIP-Enrich we also offer 'Exons': peaks are assigned to gene exons, ignoring all peaks outside of an exon. Users may also supply their own custom locus definition and/or mappability file. This enables users to study functional binding patterns relative to alternative gene features (e.g. 3′UTRs; untranslated regions) or at different distances from TSSs and to use different estimates of the observable region for each gene locus. Diagnostic plots are available to visualize the relationship between locus length and proportion of genes with a peak and to examine the proportion of peaks binding proximal or distal to TSSs. We also offer an ENCODE ChIP-Enrich Results website (http://chip-enrich.med.umich.edu/summaryReport.jsp), where users can download enrichment testing results for individual DBPs or in bulk for the GM12878 and K562 cell lines.

### FET for gene set enrichment testing of ChIP-seq data

For each GO term, we tested for association of peak presence and GO term membership using a two-sided FET. For inclusion in the analysis, genes had to be annotated in GO and have a locus defined.

### Binomial test for GO term enrichment testing of ChIP-seq data

We used a slight modification of the one-sided binomial test for GO term enrichment described by Taher and Ovcharenko (18) and implemented in GREAT (12). We calculate the one-sided probability of seeing greater than or equal to the number of peaks we observe for a GO term, *j*, with the following formula:

(4) \begin{equation*}
\sum\nolimits_{i = k_j }^n {\left( {\begin{array}{*{20}c} n \\ i \\ \end{array}} \right)} p_j^i (1 - p_j )^{n - i} ,
\end{equation*}

where *n* is the total number of peaks within gene loci present in any GO term and *k_j_* is the number of peaks annotated to GO term *j*. We define *p_j_* as the expected proportion of peaks in GO term *j*, as the total non-gapped length of the gene loci in the GO term, divided by the total non-gapped length of loci with ≥1 GO term annotation. *P*-values are calculated as the probability of observing *k_j_* or greater number of peaks in the GO term. Our implementation is consistent with other GO term enrichment programs that restrict the background gene set to those annotated in GO (33). In contrast, GREAT uses the total non-gapped genome as the denominator for *p_j_* and defines *n* as all observed peaks.

### Permutations to create ENCODE ChIP-seq data with no biological enrichment

**Figure 1.**
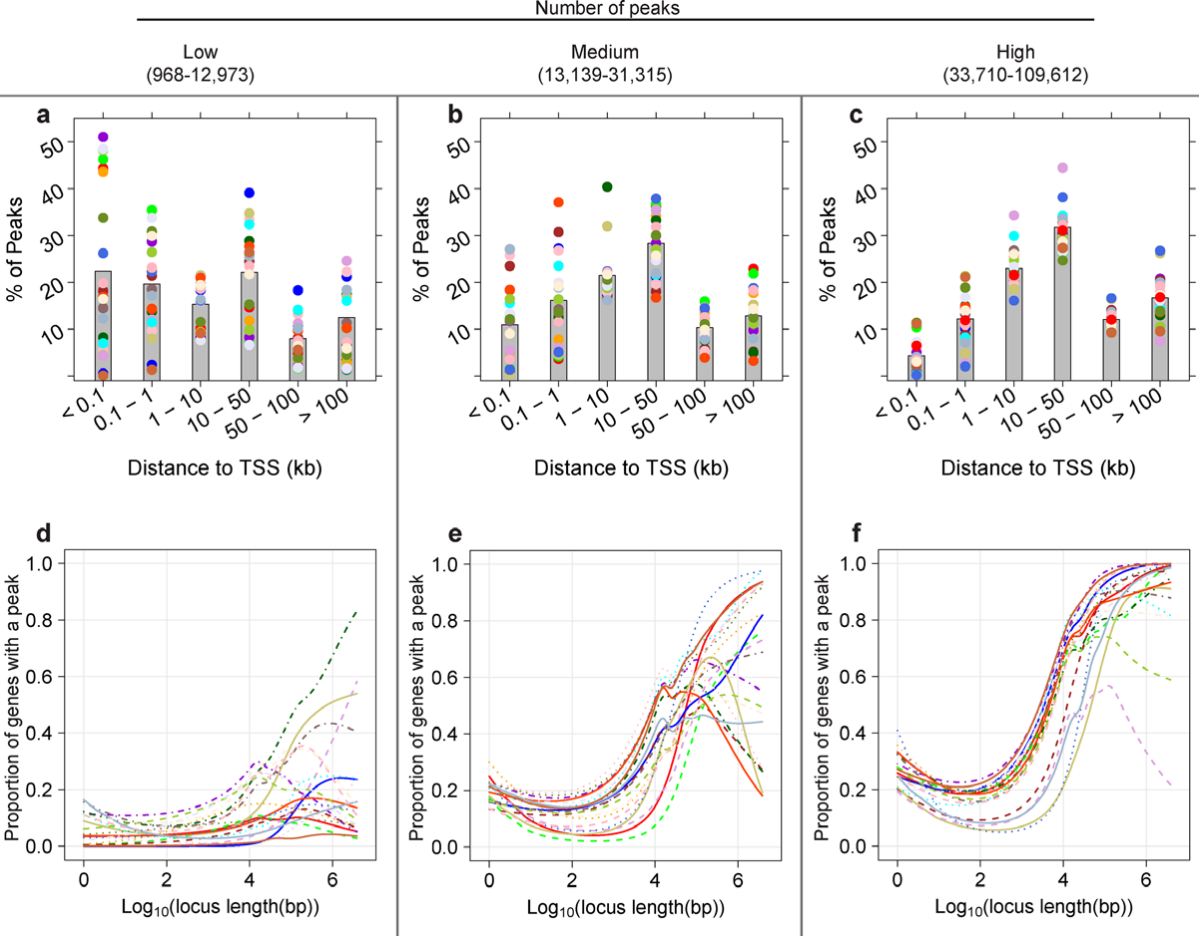
Gene locus length-to-peak presence relationship becomes stronger as the total number of peaks increases. The relationship between gene locus length and proportion of genes with ≥1 peak in a gene locus varies widely in 63 ENCODE ChIP-seq data sets, from no relationship to strongly positive. DNA-binding proteins (DBPs) from the GM12878 cell line were categorized into three groups of 21 DBPs by the total number of peaks. For each DBP, the relationship between log_10_ locus length and proportion of genes with a peak was modeled using a binomial cubic smoothing spline (see the Materials and Methods section). (**a**)–(**c**) Bar plots show the average proportion of peaks present within the specified distance from the TSS (kb) (gray bar) and the proportions for individual DBPs (colored dots, the same color as the line in the corresponding plot). DBPs with fewer peaks tend to have a higher proportion of binding close to TSSs. (**d**) The locus length-to peak presence relationship tends to be weak for data sets with few peaks. (**e**) and (**f**) The relationship becomes strongest when the number of peaks is highest (f). None of the DBPs in (d), two of the DBPs in (e) and 10 of the DBPs in (f) are histone modifications.

We performed permutations to assess the behavior of each enrichment test under two null scenarios of no true enrichment. For both scenarios, we used three ENCODE ChIP-seq data sets from cell line GM12878: SIX5 (Figure 1a and d), PAX5 (Figure 1b and e) and H3K27me3 (Figure 1c and f). For each of the two permutation scenarios below, we perform 300 permutations and test each permuted data set for GO term enrichment (5519 GO terms) using the three tests (ChIP-Enrich, FET and the binomial test).

Under both permutation scenarios, we do not expect to detect enrichment, as we have removed any association between gene membership in GO terms and the count of peaks. To help visualize the two scenarios, consider a data table, where each row represents a gene and contains the following columns: count of peaks per gene, locus length of each gene and one column for each GO term containing a (0,1) indicator variable for whether the gene belongs to that GO term. In the ‘GO term permutations’ scenario, we randomly permute the count of peaks per gene and the locus length as a unit. This results in a data set where genes (identified by their peak count and locus length) have been reassigned to new GO terms and the locus length bias inherent in GO terms has been removed, but the number of genes per GO term, correlations between GO terms and the relationship between locus length and count of peaks have all been preserved. In the ‘GO term permutation by locus length bin’ scenario, we first order the data by locus length and then randomly permute peak count and locus length as a unit, but restrict this permutation within successive bins of gene locus length (100 genes per bin). This is similar to the first scenario, but preserves the relationship between locus length and GO term membership.

### GRα analysis

We applied ChIP-Enrich to ChIP-seq peaks for GRα data from the A549 cell line from Reddy *et al*. (47): ChIP-seq peaks with FDR < 0.02 (4392 peaks). In (47), sequence reads of 36-mer length were generated from Illumina GA1, aligned using ELAND, and peaks were called using MACS. Reddy *et al*. (47) identified 209 genes as differentially expressed based on RNA-seq data from A549 cells that were treated for 1 h with 100 mM of Dexamethasone (DEX) or with 0.02% Ethanol control (EtOH). Briefly, in (47), gene expression levels were estimated using ERANGE to calculate reads per kilobase per million tags sequenced (RPKM) values, which were then adjusted for dependence of variance on expression level. A custom maximum likelihood approach was used to calculate *P*-values for the observed change in gene expression between DEX-treated and ethanol-treated cells. Finally, genes with False Discovery Rate (FDR) < 0.05 were called significant (34). Using the 209 reported differentially expressed genes, we tested for GO term enrichment (over-representation) with the R package *goseq* (19). For Table 3, we pruned the list of top-ranked, enriched GO terms of closely related terms for presentation by removing terms whose parents, children or siblings in the ontology tree were present at a higher rank in the list. We used the R package *GO.db* to determine relationships among GO terms.

## RESULTS

### Observed relationship between gene locus length and presence of at least one peak in ENCODE ChIP-seq data sets

We first explore the relationship between gene locus length and the presence of a peak in 63 ENCODE ChIP-seq data sets from tier 1 cell line GM12878 (22,35) using a binomial cubic smoothing spline to model the relationship (see the Experimental ChIP-seq peak data sets and ChIP-Enrich method sections of the Materials and Methods section) (28,29). GM12878 is a lymphoblastoid cell line, transformed using Epstein-Barr Virus, and which has a normal karyotype. Lymphoblasts are immature cells that typically differentiate into lymphocytes and serve as a good model for functional studies as they are known to express a wide range of metabolic pathways (36). This exploration of ChIP-seq data is motivated by the opposing assumptions underlying FET and the binomial test: for FET that there is no association between locus length and presence of a peak, and for binomial-based tests that the number of peaks per locus is proportional to locus length. In Figure 1, we assigned peaks to the gene with the nearest TSS (see the Materials and Methods section) and grouped the ENCODE data sets based on the total number of peaks (three equal sized groups). For data sets with the smallest number of peaks, we noticed that a large fraction of peaks were close to a TSS, and most DBPs showed no or little relationship between locus length and probability of a peak (Figure 1a and d; *n* = 21) which is consistent with the assumptions of FET, although a few showed a moderate relationship. All were transcription factor data sets. In contrast, data sets with the largest number of peaks tended to have the smallest proportion of peaks within 1 kb of a TSS and had a strong positive locus length-to-peak presence relationship (Figure 1c and f; *n* = 21), which is potentially consistent with the assumptions of the binomial test. Many of these data sets were histone modifications that tend to occur much more widely across the genome than TF binding. The locus length-to-peak presence patterns within data sets with intermediate numbers of peaks show larger variability and are often not consistent with either FET or the binomial test assumptions (Figure 1b and e).

The binomial test sums the peaks over all the genes/loci in a gene set. This summation assumes that the underlying probability of a peak per unit length is the same for each gene in the gene set (and the same for each gene not in the gene set), i.e. the variance of peak counts among genes in a gene set is no greater than expected based on the binomial distribution. We tested for variability greater than that of the binomial distribution, in GO terms containing between 50 and 500 genes. All DBPs showed a substantial proportion of GO terms with significantly (FDR < 0.05) higher variability than expected, with many DBPs having over 99% of GO terms with significant extra variability (Supplementary Table S2) (see the overdispersion test in the Materials and Methods section). Thus, even DBPs that have a strong positive relationship between the number of peaks and locus length (Figure 1f) do not satisfy the binomial test assumptions.

### ChIP-Enrich method

**Figure 2.**
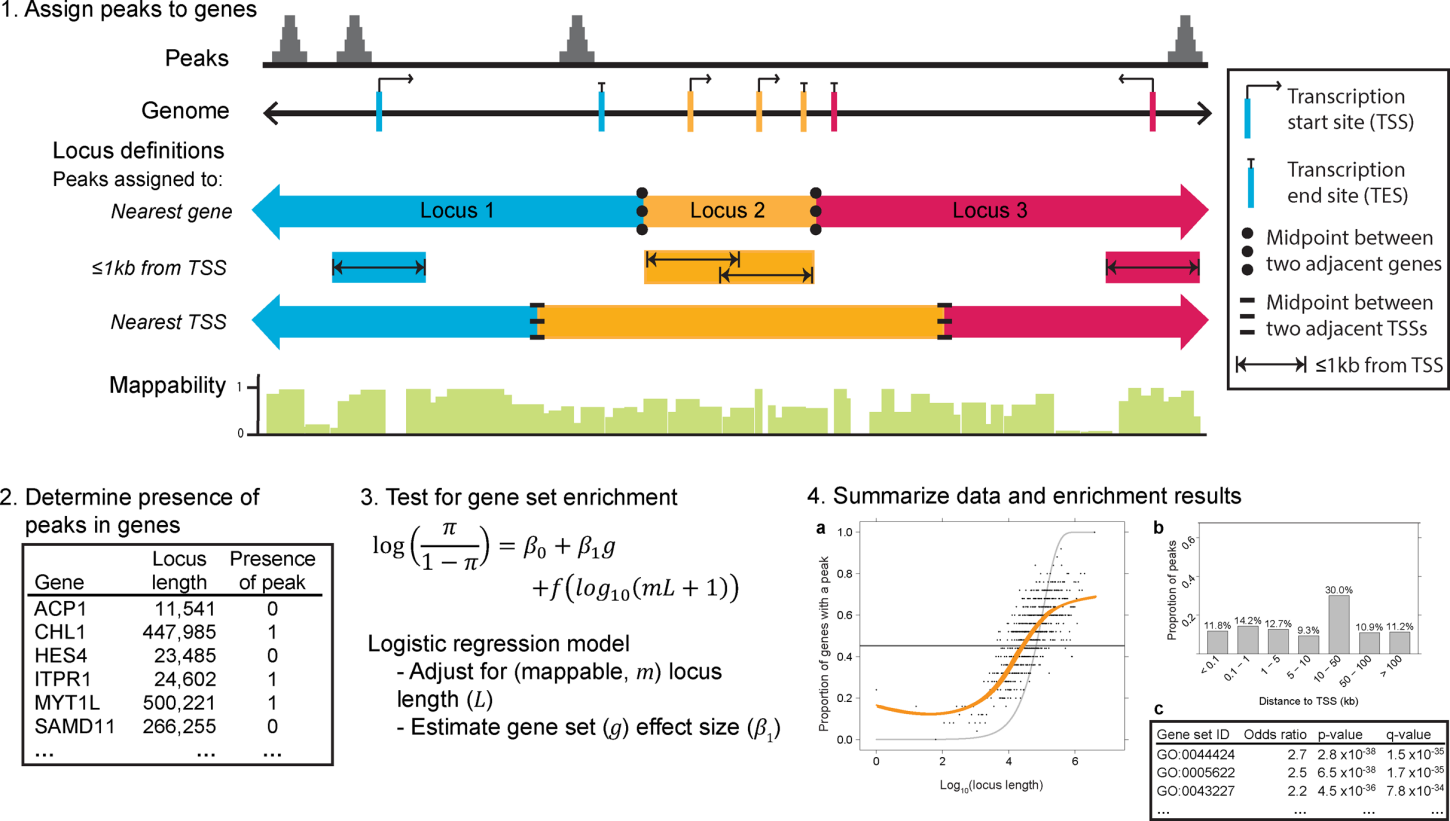
Overview of ChIP-Enrich. We describe ChIP-Enrich in four steps. (1) ChIP-seq peaks are assigned to genes using a chosen gene locus definition. Definitions include: ‘nearest gene’, ‘≤1kb from TSS’ and ‘nearest TSS’. (2) It is determined whether ≥1 peak is present in each gene locus. (3) Gene set enrichment is performed for each gene set using a logistic regression model, adjusting for locus length with a binomial cubic smoothing spline term (represented as *f* in the model equation). (4) Data and results are summarized. (**a**) Plot of observed spline fit for log_10_ locus length versus proportion of genes with a peak (orange). Expected line if no relationship between log_10_ locus length and proportion of genes with a peak (dark gray, satisfies Fisher's exact test assumptions). Expected line if the number of peaks observed is proportional to locus length (light gray, binomial test assumption). For visualization only, each point is the proportion of genes that are assigned a peak within sequential bins of 25 genes. (**b**) Bar plot of the proportion of peaks found at various distances from the TSS. (**c**) Abbreviated ChIP-Enrich output.

Given the observed locus length-to-peak presence relationships, we sought to develop a gene set enrichment testing approach for ChIP-seq data that would empirically model this relationship (Figure 2). To capture different aspects of the underlying regulatory biology, we define gene loci based on one or more genomic features and assign peaks in the defined genomic regions to genes (locus definitions). In this paper, we use as primary locus definitions: (i) the region(s) within 1 kb of every TSS of a gene (≤1 kb from TSS), (ii) the region between the upstream and downstream midpoints between a gene's TSS and the adjacent genes’ TSSs (nearest TSS) and (iii) the gene and half the intergenic region between adjacent genes, defined by the closest TSS/TES of each gene (nearest gene) (see the Gene loci definitions section of the Materials and Methods section for more details). Consistent with previous observations (20), genes with long locus lengths defined by the nearest TSS definition were significantly enriched for neuronal processes, development and adhesion (Supplementary Table S3), while genes with short locus lengths were enriched for translation and chromatin-related processes (Supplementary Table S4).

We test for gene set enrichment using a logistic regression model and adjust for the probability of a peak as a function of log_10_(observable locus length) using a binomial cubic smoothing spline (see the ChIP-Enrich method section of the Materials and Methods section). Since a logistic regression model without the smoothing spline term approximately corresponds to FET, our model is motivated by FET while accounting for locus length. Because we observed that the average mappability of gene loci both differed substantially among genes and that many GO terms were enriched with highly or lowly mappable genes (Supplementary text and Supplementary Figure S1), we also account for the average mappability of each gene locus. We calculate the proportion of each locus length that is uniquely mappable as the mappability score and use locus length × mappability as an estimate of the observable locus length (see the Mappability calculations section of the Materials and Methods section). Although mappability often improved the spline fit (Supplementary Figure S2), it had little effect on the results of these analyses. Our R package and web-based tool offer a number of additional options, including custom locus and mappability definitions (see the R package and website section of the Materials and Methods section). Thirteen gene annotation databases (32) are available for testing; for simplicity, we use GO terms to illustrate our method in our analyses below (see the GO terms section of the Materials and Methods section).

### Comparison of ChIP-Enrich, FET and the binomial test for permuted and non-permuted ENCODE data sets

To illustrate the performance of the different tests, we selected three ENCODE GM12878 DBPs with different locus length-to-peak presence relationships: SIX homeobox 5 (SIX5) (weak relationship, Figure 1d), paired box 5 (PAX5) (moderate positive relationship, Figure 1e) and trimethylation of histone 3 lysine 27 (H3K27me3) (strong positive relationship, Figure 1f) (Supplementary Figure S3). These DBPs have 75, 26 and 5% of peaks ≤1 kb from a TSS (Figure 1a–c) and 4442, 19 618 and 41 464 total peaks, respectively. We first tested for GO term enrichment with FET, the binomial test and ChIP-Enrich in the original data (see the Materials and Methods section for implementation details of FET and the binomial test). The top-ranked terms from the three tests were highly different for H3K27me3, moderately different for PAX5 and similar for SIX5 where several very strongly enriched GO terms were identified by all tests (Table 1). However, other data sets with total peaks counts similar to *SIX5 (*few peaks) (Figure 1a and d) had less agreement between the top-ranked terms for ChIP-Enrich and the binomial test (data not shown).

**Table 1.** Comparison of top five ranked GO terms for three DBPs from cell line GM12878 using ChIP-Enrich, FET and the binomial test

CE rank	Binom rank	FET rank	GO term	CE *q*-value	Binom *q*-value	FET *q*-value	Percentile*
(a) H3K27me3							
1	898	1	Extracellular matrix	1.5 × 10^−9^	0.013	2.2 × 10^−20^	69.6
2	14	4	Regulation of hormone levels	3.3 × 10^−7^	4.4 × 10^−16^	3.9 × 10^−13^	58
3	1633	3	Proteinaceous extracellular matrix	3.8 × 10^−7^	0.15	9.2 × 10^−17^	70.4
4	648	311	Cytokine activity	2.7 × 10^−6^	2.6 × 10^−3^	1.2 × 10^−3^	20.9
5	1137	122	Anchored to membrane	2.9 × 10^−6^	0.036	1.6 × 10^−5^	88.2
691	1	1066	3′,5′-cyclic-GMP phosphodiesterase activity	0.28	9.8 × 10^−32^	0.089	52.1
986	2	3715	IgG binding	0.41	1.9 × 10^−26^	0.77	1.8
256	3	2696	Pancreatic ribonuclease activity	0.095	1.10 × 10^−24^	0.77	0.1
3537	4	3186	Cytoplasmic dynein complex	0.87	9.28 × 10^−23^	1	37.4
2842	5	3049	Localization within membrane	0.99	2.6 × 10^−21^	0.92	42
14	4946	2	Synapse	1.7 × 10^−4^	1.0	3.6 × 10^−17^	91.6
21	1250	5	Sensory organ development	8.7 × 10^−4^	0.053	4.6 × 10^−13^	77.8
*Average locus length percentile for the top 20 terms for H3K27me3 for ChIP-Enrich: 59.1; binomial test: 41.6; FET: 82.2.							

(b) PAX5							
1	6	2	Immune response-regulating signaling pathway	1.4 × 10^−7^	1.1 × 10^−53^	4.5 × 10^−10^	39.6
2	4	1	Immune response-activating signal transduction	1.5 × 10^−7^	3.0 × 10^−54^	4.5 × 10^−10^	39.2
3	111	13	Protein localization to organelle	2.8 × 10^−7^	9.0 × 10^−17^	4.8 × 10^−6^	27
4	13	66	Viral reproduction	3.2 × 10^−7^	1.4 × 10^−41^	5.8 × 10^−4^	9.3
5	3	3	Leukocyte activation	5.8 × 10^−7^	1.3 × 10^−54^	5.0 × 10^−9^	48.9
20	1	39	Regulation of immune response	8.6 × 10^−5^	5.2 × 10^−74^	1.1 × 10^−4^	28.5
170	2	405	Innate immune response	0.024	4.2 × 10^−61^	0.10	20.9
49	5	31	Induction of apoptosis	5.2 × 10^−4^	3.3 × 10^−54^	8.6 × 10^−5^	30.9
6	11	4	Lymphocyte activation	1.3 × 10^−6^	5.5 × 10^−44^	9.8 × 10^−9^	52
8	19	5	Immune response-activating cell surface receptor signaling pathway	7.1 × 10^−6^	8.7 × 10^−37^	4.8 × 10^−8^	46.7
*Average locus length percentile for the top 20 terms for PAX5 for ChIP-Enrich: 25.9; binomial test: 33.3; FET: 48.6.							

(c) SIX5							
1	1	1	Ribosome	4.4 × 10^−32^	1.5 × 10^−60^	1.9 × 10^−34^	3.4
2	4	2	Structural constituent of ribosome	9.8 × 10^−25^	4.2 × 10^−49^	1.7 × 10^−27^	3.1
3	6	4	Establishment of protein localization to organelle	2.6 × 10^−23^	1.1 × 10^−43^	8.4 × 10^−24^	8.6
4	28	6	mRNA processing	6.2 × 10^−23^	5.8 × 10^−26^	2.3 × 10^−22^	22.7
5	3	5	ncRNA metabolic process	1.0 × 10^−22^	1.0 × 10^−49^	8.8 × 10^−23^	6.6
6	2	6	Viral reproduction	1.1 × 10^−22^	1.0 × 10^−52^	2.3 × 10^−22^	9.3
7	5	3	Ribosomal subunit	2.5 × 10^−22^	1.0 × 10^−46^	2.9 × 10^−24^	2.6
*Average locus length percentile for the top 20 terms for X5 for ChIP-Enrich: 8.4; binomial test: 5.1; FET: 8.0.							

(a) H3K27me3, (b) PAX5 and (c) SIX5. The most extreme differences are observed for H3K27me3, which also had the highest type I error rate for the binomial test. Differences among the tests are more moderate for PAX5. SIX5 had several extremely significant GO terms with ChIP-Enrich, which were also easily detected by the other two methods. All tests were performed using the ‘nearest TSS’ locus definition. CE: ChIP-Enrich; Binom: binomial test; FET: Fisher's exact test.

**Table 2.** FET and the binomial test, but not ChIP-Enrich, show strongly inflated type I error rates

		α level = 0.05			α level = 0.001			α level = 10^−4^		
		CE	Binom	FET	CE	Binom	FET	CE	Binom	FET
Permuted across all gene	SIX5	0.038	0.11	0.038	6.2 × 10^−4^	0.012	5.9 × 10^−4^	6.5 × 10^−5^	.0033	6.4 × 10^−5^
	PAX5	0.043	0.25	0.040	4.3 × 10^−4^	0.093	7.8 × 10^−4^	2.8 × 10^−5^	0.054	6.9 × 10^−5^
	H3k27me3	0.045	0.30	0.040	4.7 × 10^−4^	0.14	7.4 × 10^−4^	3.9 × 10^−5^	0.096	5.1 × 10^−5^

Permuted within locus length bins	SIX5	0.038	0.13	0.039	7.4 × 10^−4^	0.034	7.3 × 10^−4^	6.7 × 10^−5^	0.019	6.2 × 10^−5^
	PAX5	0.043	0.25	0.073	3.9 × 10^−4^	0.11	0.0046	3.4 × 10^−5^	0.073	0.0011
	H3k27me3	0.044	0.32	0.18	4.2 × 10^−4^	0.17	0.044	3.1 × 10^−5^	0.12	0.024

ChIP-Enrich shows the expected type I error rate in permuted ENCODE GM12878 ChIP-seq data; Fisher's exact test and the binomial test can show substantial inflation of type I error rate. Values represent the proportion of tests with *P*-value less than the given α level. For both permutation scenarios (permuted overall and permuted in locus length bins), a well-calibrated test should have type I error rate approximately equal to the α level. The total number of tests was 300 permutations × 5519 GO terms = 1 655 700 tests. CE: ChIP-Enrich; Binom: binomial test; FET: Fisher's exact test.

**Table 3.** Most significant GO terms from GRα ChIP-Enrich analysis using ‘nearest TSS’ and ‘≤1 kb from TSS’ locus definitions show a large degree of overlap with significant GO terms from RNA-seq data from the same cell line

			ChIP-Enrich *q*-value		
CE rank nearest TSS	RNA-seq rank	GO term	Nearest TSS	≤1 kb from TSS	GOseq *q*-value
(a)					
1	22	Epithelial cell differentiation	1.8 × 10^−6^	1.0	1.2 × 10^−6^
2	936	Adherens junction	5.3 × 10^−5^	1.0	0.39
4	85	Negative regulation of sequence-specific DNA binding transcription factor activity	5.5 × 10^−5^	1.0	3.0 × 10^−4^
5	9	Anti-apoptosis	5.5 × 10^−5^	0.34	3.2 × 10^−9^
7	1040	Basolateral plasma membrane	1.7 × 10^−4^	1.0	0.52
8	501	Unsaturated fatty acid metabolic process	3.2 × 10^−4^	0.028	0.063
10	872	Focal adhesion	4.5 × 10^−4^	1.0	0.32
13	132	Regulation of small GTPase-mediated signal transduction	8.6 × 10^−4^	1.0	1.3 × 10^−3^
14	95	Response to inorganic substance	1.2 × 10^−3^	0.075	4.3 × 10^−4^
15	1616	Response to growth hormone stimulus	1.4 × 10^−3^	1.0	1.0

			ChIP-Enrich *q*-value		
CE rank ≤1 kb from TSS	RNA-seq rank	GO Term	≤1 kb from TSS	Nearest TSS	GOseq *q*-value
(b)					
1	267	Negative regulation of blood coagulation	3.2 × 10^−7^	0.077	0.010
7	1143	Intrinsic to external side of plasma membrane	1.8 × 10^−4^	0.062	0.68
8	1648	Leukotriene metabolic process	2.2 × 10^−4^	6.4 × 10^−3^	1.0
10	4193	Anchored to plasma membrane	2.1 × 10^−3^	0.39	1.0
14	323	Positive regulation of leukocyte chemotaxis	3.5 × 10^−3^	0.092	0.017
15	1091	Platelet alpha granule lumen	4.7 × 10^−3^	0.25	0.61
18	1099	Ameboidal cell migration	5.2 × 10^−3^	0.31	0.94
19	1108	Regulation of nuclease activity	5.2 × 10^−3^	0.083	0.66
20	192	Cellular response to biotic stimulus	5.2 × 10^−3^	6.1 × 10^−3^	3.7 × 10^−3^
22	876	Nucleotide-binding domain, leucine-rich repeat containing receptor signaling pathway	6.1 × 10^−3^	0.15	0.010

Most highly significant GO terms (after collapsing related terms; *q*-value ≤ 0.05) detected using ChIP-Enrich with the (a) ‘nearest TSS’ and (b) ‘≤1 kb from TSS’ locus definitions. The highest ranked GO term from each related set of GO terms is displayed. Bold rows designate GO terms with *q*-value ≤0.05 in GOseq analysis of RNA-seq data. In total, 458 GO terms (with ≤500 genes) were significantly enriched for the RNA-seq results.

Under the null hypothesis of no true gene set enrichment, the type I error rate for a data set at a given threshold α is the proportion of gene sets with *P*-value less than α. A method with type I error rate higher than the expected α level will have an increased number of false positives. Therefore, we investigated the type I error rates for ChIP-Enrich, the binomial test and FET. We assessed the type I error rate using two permutation scenarios that preserve the GO term correlation structure but under which no biological enrichment exists, and therefore none should be detected. In the first scenario, we grouped gene locus length and gene peak count and permuted them together across all genes, which removes any relationship between GO term membership and locus length (permutations across all genes). In the second scenario, we grouped locus length and gene peak count and permuted them together within bins of 100 genes ordered by locus length, which retains the GO term–locus length relationship (permutations within locus length bins) (see ‘permutations’ methods in the Materials and Methods section).

In the permutations across all genes, ChIP-Enrich and FET showed slightly conservative type I error for both permutation scenarios at α = 0.05 and 0.001 (Table 2 and Supplementary text), with the slight deflation expected due to the discrete nature of the data (37). The lack of inflation for FET was expected since this permutation breaks the GO term–locus length relationship. In contrast, the binomial test had very high type I error rates at all three tested alpha levels (Table 2).

For the permutations within locus length bins, ChIP-Enrich again had the expected type I error rate (Table 2). FET showed inflation of type I error rates for PAX5 and H3K27me3, but not for SIX5. SIX5 shows little relationship between locus length and peak presence, and therefore the assumptions for FET are approximately satisfied. As a check of the ChIP-Enrich method, we compared the –log_10_(*P*-values) in the original SIX5 data and found they were highly correlated between ChIP-Enrich and FET (Pearson's *r* = 0.97), illustrating that in this case ChIP-Enrich closely approximates FET. The binomial test again had very high type I error rates for every DBP, with particularly high error for H3k27me3 (minimum permuted *P*-value = 1 × 10^−57^). Using the binomial test we observed 761 gene sets with *P* < 0.001 in the original H3k27me3 data, compared to a median of 618 for the permutated data, implying that most of the significant results for the original H3k27me3 data are false positives. For SIX5 using permutations within locus length bin, >75% of gene sets with short average locus lengths had *P*-values <0.05 with the binomial test, whereas nearly all the gene sets with long average locus lengths had *P*-values >0.9. The binomial model assumes that genes with longer locus length will have proportionally more peaks, which is not satisfied in the SIX5 data (Supplementary Figure S4a). We observed the same behavior using the GREAT program (Supplementary text and Supplementary Figure S5), but not for ChIP-Enrich (Supplementary Figure S4b). To see whether the bias in ranks based on locus length for the binomial test carried over from the permuted to the original unpermuted data, we asked if the ranks for original and permuted SIX5 data sets were correlated. We observed a high correlation for the binomial test (*r* = 0.71) between the ranks of results from the original SIX5 data and the average ranks from permutations within locus length bins, but not for permutations across all genes (Supplementary Figure S6a and b), indicating that the correlation is due to locus length. With ChIP-Enrich, there was no correlation between ranks of the original and permuted data (*r* = −0.02) as expected (Supplementary Figure S6c and d).

To complement our permutation study, we also simulated ChIP-seq peak data sets with no true biological enrichment under various scenarios and tested for enrichment with ChIP-Enrich, the binomial test and FET. In these simulations, the binomial test had an inflated type I error rate when peak counts were not proportional to locus length or when extra variability (overdispersion) was added to gene peak counts. Only ChIP-Enrich showed the expected type I error rate in all simulations (Supplementary text and Supplementary Figures S7 and S8).

### Influence of locus definition on detection of gene set enrichment

For each of the 63 GM12878 ChIP-seq data sets, we asked if dissimilar sets of biologically related genes were detected using different locus definitions, as a way to identify DBPs that regulate distinct biological functions from different regulatory regions. Comparing ChIP-Enrich results for peaks assigned to the ‘nearest TSS’ to those of the ‘nearest gene’, we found moderate to high correlations in the enrichment results (Pearson's *r* = 0.62–0.99 for –log_10_ *P*-values) and *P*-values of similar magnitude, indicating that the two definitions are capturing similar information.

**Figure 3.**
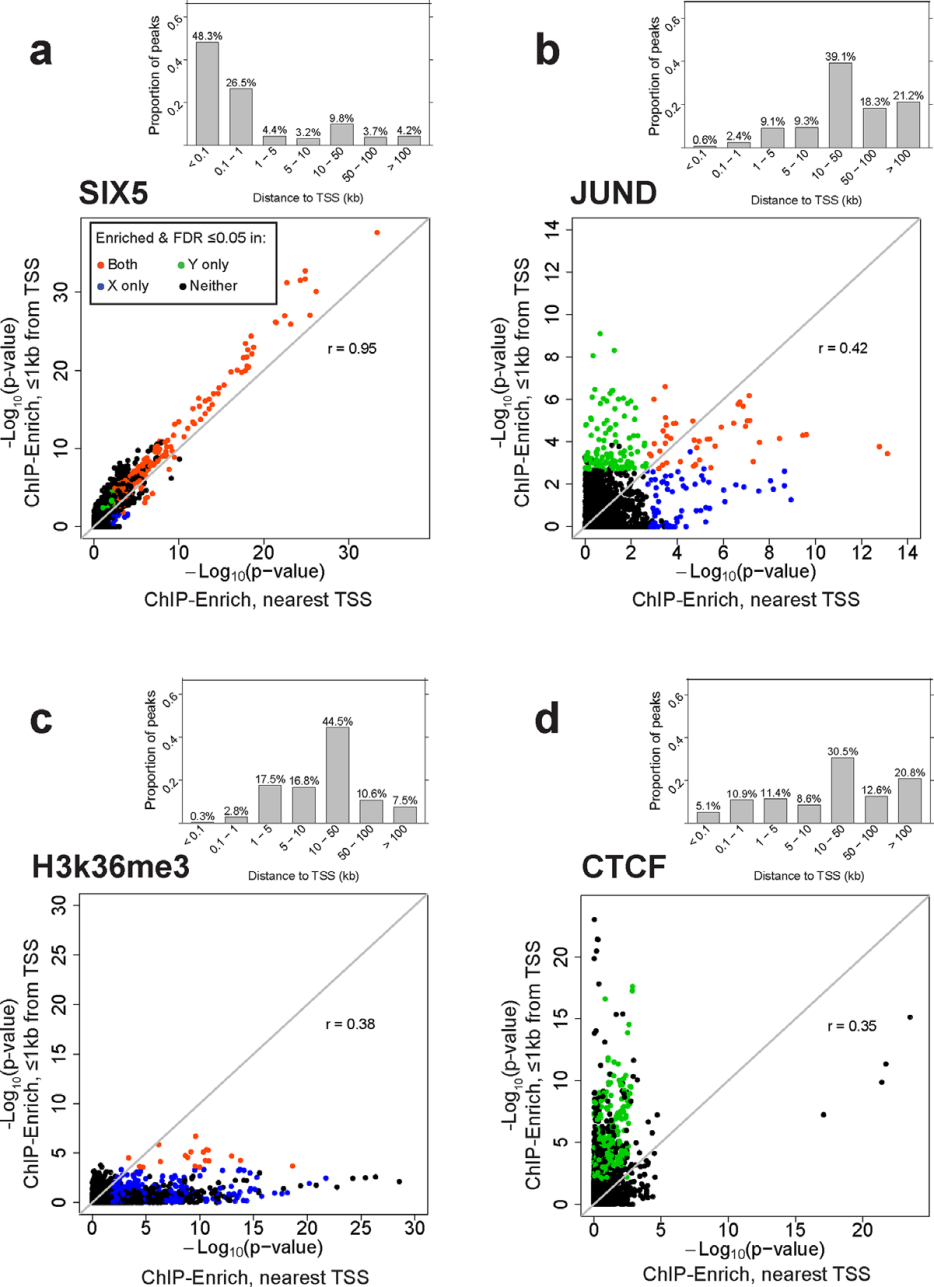
Representative plots of the four patterns of enrichment comparing the ‘≤1kb from TSS and nearest TSS’ locus definitions. Gene set enrichment testing using the ‘≤1kb from TSS’ and ‘nearest TSS’ locus definitions may identify similar (**a**) or different (**b**) sets of significant GO terms for the same DBP. Alternatively, most of the enrichment signal may come from ‘nearest TSS’ that uses all peaks (**c**) or ‘≤1 kb from TSS’ that ignores peaks >1 kb from a TSS (**d**). (a)–(d) Upper plot: bar plot of the proportion of peaks at different distances from the TSS. Lower plot: comparison of –log_10_(*P*-values) from ChIP-Enrich GO term enrichment testing using ‘≤1 kb from TSS’ versus ‘nearest TSS’ locus definitions in ENCODE data for the GM12878 cell line. GO terms enriched with FDR ≤ 0.05 for: ‘≤1 kb from TSS’ only (green); ‘nearest TSS’ only (blue); ‘≤1 kb from TSS’ and ‘nearest TSS’ (orange); neither analysis (black). *r*, Pearson correlation coefficient. These patterns are representative of patterns present in 63 ENCODE DBPs from the GM12878 cell line.

We observed much greater variability in comparisons between the ‘≤1 kb from TSS’ and ‘nearest TSS’ locus definitions, with four distinct patterns emerging (Figure 3 and Supplementary Figure S9). (i) We found similar results for ‘≤1 kb from TSS’ and ‘nearest TSS’ for DBPs that tend to bind near TSSs, such as SIX5 (Figure 3a), and for a subset of other DBPs (Supplementary Figure S9). (ii) We identified distinct GO terms for ‘≤1 kb from TSS’ and ‘nearest TSS’ for JunD and a small number of other DBPs (Figure 3b). JunD showed strong enrichment for calcium ion-related terms only within 1 kb of a TSS and enrichment for the JNK (Jun N-terminal kinase) and MAPK (Mitogen-activated protein kinase) cascades only using ‘nearest TSS’ (not shown). JunD regulates varied physiological processes (38); these results suggest it may regulate different processes from near versus far TSSs. (iii) We identified much stronger enrichment using ‘nearest TSS’ than ‘≤1 kb from TSS’ for H3K36me3 (Figure 3c), H3k79me2 and H4k20me1 (Supplementary Figure S9) which bind along gene bodies (39). (iv) Finally, we saw much stronger GO term enrichment using ‘≤1 kb from TSS’ than using ‘nearest TSS’ for CTCF (Figure 3d), WHIP and a subset of DBPs with a small percent of peaks ≤1 kb from the TSS (Supplementary Figure S9).

Although CTCF is a well-known insulator in intergenic regions, both CTCF binding and housekeeping genes are enriched in the boundary regions of genomic topological domains (40), and we see many of the same strongly enriched GO terms for CTCF binding ≤1 kb from a TSS (RNA processing, mitochondrion and cell cycle) as for genes identified at the boundary regions. WHIP binds to damaged DNA and in that capacity is not expected to bind within or near genes with specific functions (41,42). The most highly enriched gene sets for WHIP using the ‘≤1 kb from TSS’ definition included DNA repair (*P* = 1.1 × 10^−17^), chromatin organization (*P* = 3.6 × 10^−15^) and cell cycle regulation suggesting transcriptional roles of WHIP related to its direct function in DNA repair. Other DBPs with relatively small percentages of peaks near a TSS also showed stronger ‘≤1 kb from TSS’ enrichment results; these have known transcriptional functions and/or involvement in DNA repair (ZNF143, CHD2) (43,44), chromatin structure (EBF1) (45) or centromere formation (SMC3) (46), which may explain the lack of biological enrichment from more distal peaks (Supplementary Figure S9).

### ChIP-Enrich analysis of the GRα

**Figure 4.**
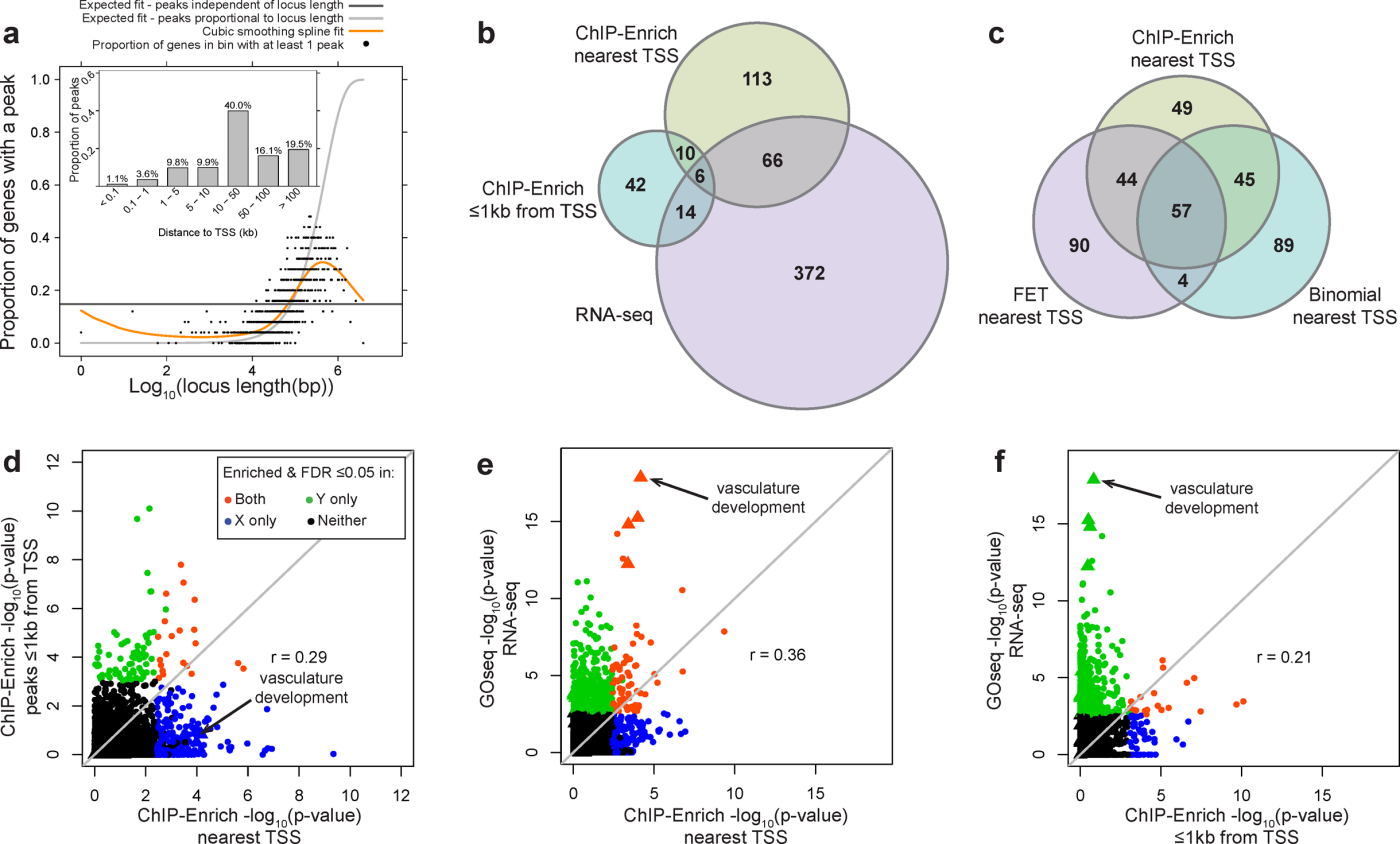
Comparison of GRα enrichment results for ChIP-seq (using two locus definitions) and RNA-seq data from A549 cells. Enriched GO terms for differentially expressed transcripts and GRα binding following 100-nM DEX treatment show stronger overlap using the ‘nearest TSS’ locus definition than using the ‘≤1 kb from TSS’ definition. (**a**) Observed spline fit for GRα fits neither FET nor the binomial test assumption (orange); bar plot of the proportion of peaks at different distances from the TSS. See Figure 2(4a) and (b) for further details. (**b**) Using the ‘nearest TSS’ locus definition with GRα results in more overlapping terms with RNA-seq results than using ‘≤1 kb from TSS’. (**c**) Using the top 195 ranked terms for each test, FET and the binomial test have more overlap with ChIP-Enrich than with each other. (**d**)–(**f**) Comparison of –log_10_(*P*-values) for GO term enrichment tests based on ChIP-seq data (ChIP-Enrich) and/or RNA-seq (GOseq) data. (f) Many enriched RNA-seq terms would have been missed in the ChIP-seq data if only peaks in promoter regions were considered. GO terms enriched and FDR ≤ 0.05: for Y-axis test only (green); for X-axis test only (blue); for X- and Y-axis tests (orange); for neither (black). Vasculature development and related GO terms (triangles). The majority of GO terms that overlap between ‘≤1 kb from TSS’ and ‘nearest TSS’ are related to fatty acid metabolism, reactive oxygen species and unfolded proteins, or blood coagulation.

We asked whether ChIP-Enrich could identify known and potential new biology of a well-characterized transcription factor, the GRα (47). Previous analysis identified 4392 peaks in A549 cells treated with 100-nM DEX (dexamethasone stimulates GR activity); only 4.7% of the peaks were within 1 kb of a TSS (Figure 4a). GO term enrichment testing yielded largely distinct subsets of significant (FDR ≤ 0.05) terms for ‘nearest TSS’ (195 terms) and ‘≤1 kb from TSS’ (72 terms) with only 16 overlapping terms (Figure 4b and d; Supplementary Table S5). The most significant terms (after collapsing similar terms) are shown in Table 3. Terms significant using one or both locus definitions include ‘epithelial cell differentiation’ (*q*-values: nearest TSS = 1.8 × 10^−6^; ≤1 kb from TSS = 1.0) and ‘negative regulation of blood coagulation’ (*q*-values: nearest TSS = 0.077; ≤1 kb from TSS = 3.19 × 10^−7^, with the related term ‘regulation of wound healing’ (*q*-values: nearest TSS = 0.0064; ≤1 kb from TSS = 0.0029). In addition, we observed ‘response to glucocorticoid stimulus’ (*q*-values: nearest TSS = 0.0035; ≤1 kb from TSS = 0.55) and ‘regulation of lipid metabolic process’ (*q*-values: nearest TSS = 0.0062; ≤1 kb from TSS = 0.74). GRα is known to be involved in the response to steroids and the activation of lipolysis (48,49), although knowledge of the transcriptional role of GRα in wound healing and blood coagulation is more limited. We also tested for enrichment using non-overlapping locus definitions for regions closer to a TSS (≤5 kb from TSS; 14.5% of peaks) and further from a TSS (>10 kb from TSS; 75.6% of peaks*)* and again identified largely distinct gene sets (Supplementary Figure S10).

We also compared the enrichment results (using ‘nearest TSS’) from ChIP-Enrich with those using the binomial test and FET. Due to inflated type I error rates for the binomial test and FET for ‘nearest TSS’, the specific *P*-values and number of terms with FDR < 0.05 cannot be used. Instead, we compared the top-ranked terms among the methods, using the number of top-ranked terms with FDR < 0.05 for ChIP-Enrich (195). There was substantial overlap, with 57 (29%) GO terms identified by all three methods and 150 (77%) identified by at least two (Figure 4c). Both FET and the binomial test had higher overlap with ChIP-Enrich than with each other, consistent with the fact that the locus length-to-peak presence relationship modeled by ChIP-Enrich is intermediate between the assumptions of FET and the binomial test.

To evaluate the biological relevance of our results, we compared the ChIP-seq enrichment results from ChIP-Enrich with RNA-seq enrichment results based on differential expression between control and 100-nm-DEX-treated A549 cells (47) (see the GRα analysis section of the Materials and Methods section). Of 4544 GO terms tested for enrichment based on RNA-seq differential expression, 458 (10%) were significant at FDR ≤ 0.05. ‘Vascular development’, the most significant GO term based on differential expression, was also significantly enriched for GRα binding using the ‘nearest TSS’ analysis (*q*-value = 0.0047) but not using ‘≤1 kb from TSS’ (*q*-value = 0.97). Eighty six (29%) of the significant terms from RNA-seq were significant with one or both of the locus definitions in ChIP-seq data (Figure 4b). From the ChIP-seq perspective, many of the most highly significant terms using ‘nearest TSS’ and ‘<1 kb from TSS’ were significant for RNA-seq (Table 3 and Figure 4e and f). Seventy two (37%) of the significant GO terms for ‘nearest TSS’ were significant for RNA-seq, whereas only 20 (28%) of the significant GO terms for ‘≤1 kb from TSS’ were significant for RNA-seq, indicating potentially stronger correspondence of the gene expression data with the GRα peaks captured by the ‘nearest TSS’ definition than only those peaks ≤1 kb from a TSS. GO terms enriched only in RNA-seq may be regulated by genes downstream of those directly regulated by GRα or be GRα-independent DEX effects. GO terms enriched only in ChIP-seq data may indicate pathways that are poised to be regulated, either from proximal promoter or more distal enhancer regions.

## DISCUSSION

We developed a gene set enrichment testing method for ChIP-seq data, ChIP-Enrich, that empirically models and adjusts for the effect of gene locus length. In contrast to Fisher's exact and the binomial test, ChIP-Enrich maintains the correct type I error rate for data sets with a wide range of locus length-to-peak presence relationships. FET and the binomial test make assumptions that are inconsistent with the observed relationships, which can lead to inflated type I error rates (false positive results). Strikingly, the binomial test often has significantly more false positives than FET.

ChIP-Enrich uses a binomial smoothing spline to empirically model the relationship with gene locus length, an approach similar to that employed by GOseq, which was developed for RNA-seq data (19). Whereas GOseq uses either a resampling approach or the approximate Wallenius method to calculate GO term enrichment *P*-values, ChIP-Enrich incorporates the smoothing spline in a logistic regression model, allowing more precise *P*-value calculations and in less time than a resampling approach requires.

For many DBPs, particularly those with more binding near TSSs, testing for enrichment using the ‘nearest TSS’ and ‘≤1 kb from TSS’ locus definitions identifies largely overlapping gene sets, suggesting the two definitions often capture similar regulatory information. However, for a subset of DBPs, these two locus definitions detect very different enriched gene sets. JunD, for example, may be regulating different biological processes nearer to and further from the TSS, possibly with different cooperating factors. For data sets with a small proportion of peaks ≤1 kb of a TSS, but stronger levels of enrichment detected with those peaks (examples WHIP and CTCF), it is possible that DBP binding >1 kb from the TSS may not be properly assigned to the regulated gene(s) or that some of the widespread DBP binding may not regulate genes in any specific biological processes. Thus for DBPs with unknown function, comparisons of patterns of gene set enrichment could help predict an alternative role for the DBP, such as DNA repair and/or chromatin remodeling or looping.

To further explore the biological relevance of our results, we compared the gene sets enriched for differential expression of messenger RNA (mRNA) following activation of GRα to the gene sets enriched for GRα binding (47). For GRα a subset of gene sets, many of which were not detected using the ‘≤1 kb from TSS’ locus definition and including vasculature development, showed substantial enrichment for both differential expression and GRα binding. GRα has been reported to play a limited role in vasculature development, mainly through non-transcription factor activities; the extent to which it directly regulates vasculature development genes as a group was thus far unknown (50–52). This suggests that GRα regulates many genes and functions via binding further from TSSs, consistent with the observations of Reddy *et al*. (47), and this regulation would be missed if only peaks within 1 kb were examined (such as could be tested without bias using FET).

Unlike the binomial test, ChIP-Enrich results are not influenced by a single gene or few genes with a large number of peaks because it does not consider the number of binding sites per gene. However, because higher numbers of a bound DBP in a gene locus may exert stronger biological effects (49), the use of a model based on peak counts per gene, which accounts for extra variability and diverse locus length-to-peak count relationships, could be considered. For example, a negative binomial or beta binomial model may be able to account for the extra-variability among genes in the peak count data. However, it is unclear whether these models can fully account for both the extra variability and the observed negative correlation between peak occurrence rate and locus length, or how best to empirically adjust for locus length.

In conclusion, we developed a gene set enrichment testing method, ChIP-Enrich, which allows enrichment analysis of ChIP-seq data with any locus length-to-peak presence relationship with the expected type I error rate. This is in contrast to currently available methods, which often exhibit highly elevated type I error and/or gene set ranking biased toward genes with long or short locus length, leading to false positive results. Based on our observations, we recommend testing each set of genomic regions for enrichment with both a locus definition representing promoter regions (e.g. ‘≤1 kb from TSS’ or ‘≤5 kb from TSS’) and a locus definition representing all regions or regions more distal to TSSs (e.g. ‘nearest TSS’, ‘nearest gene’, or ‘>10 kb from TSS’). ChIP-Enrich can be used to further assess and refine regulatory region definitions, based on empirical exploration, and to identify biological functions of regions exhibiting various complex patterns of histone marks or protein binding using the wealth of biological data from ENCODE, the Roadmap Epigenomics Program and other public and non-public sources. With the option for user-defined locus definitions and/or mappability tracks, this framework can also be used with other genome-wide sequencing data such as RNA-seq (with potential bias from transcript length and/or read depth) or bisulfite sequencing data (with potential bias from the number of measured CpG sites).

## SUPPLEMENTARY DATA

Supplementary Data are available at NAR Online, including [1–8].

SUPPLEMENTARY DATA
