# Supplementary material for: ChIP-Enrich: gene set enrichment testing for ChIP-seq data
Source: Nucleic Acids Res. 2014 May 30;42(13):e105. doi: 10.1093/nar/gku463 (PMC4117744; doi:10.1093/nar/gku463)
Supplement: SUPPLEMENTARY DATA [file supp_42_13_e105__index.html]

ChIP-Enrich: gene set enrichment testing for ChIP-seq data — SUPPLEMENTARY DATA 

# ChIP-Enrich: gene set enrichment testing for ChIP-seq data

## SUPPLEMENTARY DATA

**Files in this Data Supplement:**

- SUPPLEMENTARY DATA
- SUPPLEMENTARY DATA
- SUPPLEMENTARY DATA
