# Supplementary material for: ChIP-Enrich: gene set enrichment testing for ChIP-seq data
Source: Nucleic Acids Res. 2014 May 30;42(13):e105. doi: 10.1093/nar/gku463 (PMC4117744; doi:10.1093/nar/gku463)
Supplement: SUPPLEMENTARY DATA [file supp_gku463_nar-00162-met-g-2014-File006.pdf]

ChIP-Enrich Supplementary Results

Effect of locus length and read mappability on gene set enrichment tests

Gene set enrichment testing with Fisher's exact test (FET) can be confounded when there is a positive relationship between locus length and the presence of a peak, since many gene sets contain genes with substantially longer or shorter locus lengths than average [1]. Taher and Ovcharenko (2009) identified Gene Ontology (GO) terms with much longer or shorter than expected gene loci (defining a locus as the gene and half the intergenic region between adjacent genes) [2]. Similarly, when we assigned peaks to the gene with the nearest TSS, we found that GO terms related to nucleosome, protein-DNA complexes, and translation have genes with shorter than average locus lengths (Supplementary Table 3) and nervous system development, cell adhesion, and transcription have genes with longer than average locus lengths (Supplementary Table 4).

The probability of calling a ChIP-seq peak can depend on the mappability of the reads in the binding region [3, 4]. To account for mappability (which similar to locus length, varied significantly by gene set (Supplementary Figure 1)), we use locus length × mappability as the observable locus length in our analyses; this can improve the spline fit (Supplementary Figure 2), although it had little effect on the final results of the presented enrichment testing analyses (*data not shown*). To assess the ability of mappability to confound the relationship between the presence of a peak and gene set membership, we estimated the average mappability of each gene locus based on base pair mappabilities for 50bp reads (see Supplementary Figure 1a for comparison of mappability at different read lengths). Genes with less mappable loci are significantly more likely to be present in sensory, xenobiotic response and oxygen related terms, whereas genes with highly mappable loci are more likely to be involved in nervous system or development terms (q-value $< 3.0 \times 10^{-16}$) (Supplementary Figure 1b,c). We observed similar results at other read lengths (*data not shown*). Several GO terms (e.g., central nervous system development) had both longer locus lengths and higher mappability, increasing the possibility for confounding.

In addition to mappability, GC content has also been noted to influence sequencability and thus detection of ChIP-seq peaks. To examine a potential bias due to GC content, we downloaded the UCSC Genome Browser's GC content track for hg19, which provides GC content for every 5bp. We calculated average GC content for four definitions of gene loci (*nearest TSS*, *exons*, *1kb* and *5kb*), and observed very little spread in the distribution of GC content. Testing for GO terms enriched with low or high GC content genes (using the *nearest TSS* definition and the same LRpath approach as used for testing low or high mappability genes), we found only 14 significant terms as compared to 717 significant terms for mappability (FDR<0.05). Given the tight distribution of GC content among gene loci and the small number of significantly associated GO terms, we conclude that it is unlikely that GC content is a confounding variable or significantly biases the enrichment testing results.

Comparison of ChIP-Enrich, Fisher's exact test, and the binomial test under the null hypothesis of no enrichment using simulated data

To examine the sensitivity of each test to varying mixtures of peak distributions that meet the FET or binomial test assumptions, we simulated datasets of 10,000 peaks with 0%, 50%, and 100% of the peaks simulated in proportion to locus length relative to those simulated irrespective of locus length. As the percentage of peaks simulated in proportion to locus length increases from 0 to 100%, the relationship

between the probability of a gene having at least one peak and locus length changes from flat (Supplementary Figure 7a) to increasingly correlated (Supplementary Figure 7b,c).

Using our simulated datasets, we tested for GO term enrichment and plotted the observed $-\log_{10}$(p-values) versus the expected $-\log_{10}$(p-values) under the null hypothesis of no enrichment in quantile-quantile (QQ) plots (Supplementary Figure 7d-f.) For all three scenarios, ChIP-Enrich shows no inflation of significance levels from the expected distribution but has a slight deflation of the most significant p-values. When all peaks are simulated with each gene having equal probability of having a peak (0% proportional to locus length), Fisher's exact test shows the expected distribution of p-values (observed = expected) also with a slight deflation of the most significant p-values similar to ChIP-Enrich, expected due to the discrete nature of the data [5] . With an increasing percentage of peaks sampled in proportion to locus length, FET becomes increasingly anti-conservative (Supplementary Figure 7d → e → f), such that p-values as low as 10^{-10} are observed in the absence of any true enrichment. The binomial test shows the opposite behavior; when peaks are sampled in proportion to locus length (100% proportional to locus length) and without any additional variability among genes in a gene set, the binomial test has the expected p-value distribution (again with a slight deflation as for FET when 0% random) but when peaks are sampled independent of locus length (0% proportional to locus length) the test becomes increasingly anti-conservative (Supplementary Figure 7f → e → d), with even lower p-values than observed for Fisher's exact test.

Test behaviors in the presence of overdispersion of peak counts among genes, given locus length

To better understand the difference in binomial test behavior between 1) the H3K27me3 dataset GO term permutation by locus length bin (which shows a strong inflation of significance levels despite peaks occurring approximately in proportion to locus length) and 2) simulations in which 100% of peaks were simulated in proportion to locus length (Supplemental Figure 8f; which shows no inflation of significance levels when peaks occur in proportion to locus length), we performed additional simulations with 100% of the peaks simulated in proportion to locus length. In these simulations, we added increasing levels of extra variability (overdispersion) in peak counts among genes (gamma variance levels of 0.01, 0.1, and 0.5). The overdispersion did not visually change the observed spline fit (*not shown*). Again, we tested for GO term enrichment and plotted the observed $-\log_{10}$(p-values) versus the expected $-\log_{10}$(p-values) in QQ plots (Supplementary Figure 8). As before, ChIP-Enrich shows no inflation of significance levels. The binomial test, however, shows increasing inflation of significance levels with increasing overdispersion. FET shows decreasing levels of inflation with increasing overdispersion, but remained biased for each overdispersion scenario.

Slight deflation in p-values compared to what is expected under the null

When the assumptions of Fisher's exact test are met, e.g. for the transcription factor SIX5, Fisher's exact test shows a slight deflation of the most significant p-values compared to what is expected if we assume a uniform distribution of p-values under the null hypothesis. This trend is expected due to the discrete nature of the data [5]. We observe the same slight deflation for both ChIP-Enrich (Figure 3c,g,k, and Supplementary Figures 7d,e,f, and 8b) and the binomial test (Supplementary Figures 7f and 8c) when the assumptions of the test are satisfied.

Sensitivity analysis for GR

As a sensitivity analysis we also repeated the GRα analyses with a larger set of peaks identified using a less stringent cutoff. This set contains 15,837 peaks with p-value ≤ 1 $x10^{-9}$, equivalent to an FDR < 0.23 (Supplementary dataset 1 from Reddy et al.) [6]. Results using *nearest TSS* with the 15,837 peaks were similar to those from the more stringent peak calling (r =0.61 for $-\log_{10}$(p-value) comparison) (see Supplementary text), with 81/216 (38%) of the significant GO terms also significant in the RNA-seq enrichment analysis. However, the *≤1kb from TSS* analysis results from the less stringent peak calling identified only 26 GO terms compared to 72 from the more stringent peak calling with only 8 GO termsin common. Only five (19%) of the 26 GO terms were also significant in the RNA-seq GOseq enrichment analysis, consistent with our finding in the main text that there is potentially stronger correspondence of gene expression data with GRα binding captured by *nearest TSS* (mainly distal regions) than only those peaks ≤1kb from a TSS.

Supplementary methods

Testing for enriched GO terms with genes of longer (or shorter) than average locus length

We used DAVID [7] to test for GO term enrichment in the top 500 genes with longest (or shortest) locus lengths. For both tests, the complete set of genes in our locus definition file was used as the background gene list. Results were limited to GO terms with ≤2,000 genes and FDR≤0.05 in order to report more specific categories.

Testing for enriched GO terms with genes having higher or lower than average mappability

We tested for GO terms enriched with genes having higher or lower than expected mappability scores using a logistic regression model with GO term membership as the outcome and average gene locus mappability scores as the predictor (LRpath [8]; lrpath.ncibi.org). Because LRpath typically accepts p-values as input which are then log-transformed, we exponentiated mappabililty values before input to preserve the original mappability scale. Results were limited to GO terms with ≤2,000 genes and FDR≤0.05.

Simulation and enrichment testing of data under the null hypothesis of no GO term enrichment

We simulated ChIP-seq peaks under the null hypothesis of no association with any GO term. As an alternative to simulating peak locations, we randomly sampled genes with replacement and set the number of times the gene was selected to the count of peaks occurring within the locus of a gene. Genes were sampled in two ways: 1) randomly (not in proportion to locus length), and 2) randomly in proportion to locus length. The first simulates peaks occurring within genes with no dependence on locus length (FET assumption). The second method simulates peaks being assigned to genes with probability in proportion to locus length (binomial test assumption). We simulated datasets of 10,000 peaks with varying percentages (0, 50 and 100%) sampled in proportion to locus length. For FET and ChIP-Enrich, a gene is labeled as having a peak if the count of peaks is ≥ 1. Each GO term was tested for enrichment using FET, the binomial test, and ChIP-Enrich. We repeated this process 1,000 times for each test and percentage of genes sampled by locus length, and calculated the median of the 1,000 simulation p-values at each quantile of the 2,565 GO term p-values to create the plots for the bottom row of Supplementary Figure 7.

To examine the effect of overdispersion (added variation in peak count among genes) on each of the three tests, we simulated data with 100% of peaks sampled by locus length (satisfying the binomial test assumption) but with additional overdispersion in number of peaks per gene. In the simulations above without overdispersion, we sampled genes in proportion to their locus lengths to represent ChIP-seq peaks

occurring in gene loci, by assigning each gene a weight proportional to its locus length. Here, we sample genes in proportion to random deviates of their locus lengths using a gamma distribution with mean equal to the gene's locus length and variance set to one of four different levels (0, 0.01, 0.1, 0.5) to simulate increasing overdispersion. For each simulation, a weight is drawn for each gene and then 10,000 draws of genes are made based on the weights to represent 10,000 peaks. For each gamma variance level, we performed 1,000 simulations. The simulated data was tested for enriched GO terms using FET, ChIP-Enrich and the binomial test, and results were presented as median quantile p-values as above (Supplementary Figure 8).

Code for all simulations is available in Supplementary_code.zip.

GREAT testing on permuted ChIP-seq datasets

To confirm that our results in Supplementary Figure 4 were not restricted to our implementation of the binomial test, we repeated the analysis of GO term permutations by locus length bins data (permuted ENCODE datasets for SIX5, PAX5, and H3k27me3) with the GREAT website (Supplementary Figure 5). For each of the three experimental datasets, we used GREAT with the "single gene" setting, where "each gene is assigned a regulatory domain that extends in both directions to the nearest gene's TSS but no more than the maximum extension in one direction." The "maximum extension" was set to 100,000 kb in order to insure each peak is assigned to a regulatory domain (i.e. gene), which is most equivalent to our *nearest TSS* locus definition.

Supplementary References

1. Ovcharenko I, Loots GG, Nobrega MA, Hardison RC, Miller W, Stubbs L: **Evolution and functional classification of vertebrate gene deserts**. *Genome Res* 2005, **15**(1):137-145.
2. Taher L, Ovcharenko I: **Variable locus length in the human genome leads to ascertainment bias in functional inference for non-coding elements**. *Bioinformatics* 2009, **25**(5):578-584.
3. Rashid NU, Giresi PG, Ibrahim JG, Sun W, Lieb JD: **ZINBA integrates local covariates with DNA-seq data to identify broad and narrow regions of enrichment, even within amplified genomic regions**. *Genome Biol* 2011, **12**(7):R67.
4. Rozowsky J, Euskirchen G, Auerbach RK, Zhang ZD, Gibson T, Bjornson R, Carriero N, Snyder M, Gerstein MB: **PeakSeq enables systematic scoring of ChIP-seq experiments relative to controls**. *Nat Biotechnol* 2009, **27**(1):66-75.
5. Upton GJG: **Fisher's Exact Test**. *Journal of the Royal Statistical Society Series A (Statistics in Society)* 1992, **155**(3):395-402.
6. Reddy TE, Pauli F, Sprouse RO, Neff NF, Newberry KM, Garabedian MJ, Myers RM: **Genomic determination of the glucocorticoid response reveals unexpected mechanisms of gene regulation**. *Genome Res* 2009, **19**(12):2163-2171.
7. Dennis G, Jr., Sherman BT, Hosack DA, Yang J, Gao W, Lane HC, Lempicki RA: **DAVID: Database for Annotation, Visualization, and Integrated Discovery**. *Genome Biol* 2003, **4**(5):3.
8. Kim JH, Karnovsky A, Mahavisno V, Weymouth T, Pande M, Dolinoy DC, Rozek LS, Sartor MA: **LRpath analysis reveals common pathways dysregulated via DNA methylation across cancer types**. *BMC genomics* 2012, **13**(1):526.

Supplementary Results: Tables

Supplementary Table 1

DNA binding protein	Peak caller	Number of peaks	% of peaks ≤ 1kb from TSS
ATF3	spp	1884	66.9
BATF	spp	24600	4.9
BCL11A	spp	13256	5.7
BCL3	MACS	22503	13.1
BCLAF1	MACS	29162	30.8
BHLHE40	MACS	57698	21.7
BRCA1	MACS	15431	40.6
C-Fos	spp	1744	81.8
CHD2	MACS	42652	29.4
CTCF	MACS	44056	16.1
Ctcf	Scripture	61525	12.8
EBF1	MACS	98976	14.2
EGR1	spp	13662	54.3
ELF1	spp	20528	52.7
ETS1	spp	2879	72.6
Ezh2	Scripture	64277	9.9
GABP	spp	5095	79.9
H2az	Scripture	95358	15.0
H3k27ac	Scripture	56069	18.5
H3k27me3	Scripture	41464	5.4
H3k36me3	Scripture	33710	3.0
H3k4me1	Scripture	109612	8.9
H3k4me2	Scripture	79675	15.3
H3k4me3	Scripture	57476	17.6
H3k79me2	Scripture	28302	13.7
H3k9ac	Scripture	41266	25.5
H3k9me3	Scripture	74515	2.3
H4k20me1	Scripture	23943	5.0
JunD	spp	1715	3.0
MAX	spp	2087	39.7
MEF2A	spp	16694	11.5
MEF2C	MACS	968	15.1
NF-E2	MACS	12973	24.2
NFKB	spp	10073	22.1
NRF1	spp	5042	12.7
NRSF	spp	2541	39.2
P300	spp	3687	16.5
PAX5	spp	19618	18.5
PBX3	spp	7431	26.1
POL2	MACS	14989	33.1
POL3	MACS	112	55.5

POU2F2	spp	14441	32.9
PU.1	spp	35821	11.3
RAD21	MACS	23947	6.5
RFX5	MACS	26336	33.4
RXRA	spp	2965	31.4
SIX5	spp	4442	74.8
SMC3	MACS	64597	14.4
SP1	spp	13139	46.1
SRF	spp	2412	48.7
STAT3	MACS	24257	15.1
TAF1	spp	5169	82.4
TBP	MACS	31315	30.4
TCF12	spp	15028	25.8
TR4	MACS	1530	29.5
USF1	spp	7074	43.0
USF2	MACS	30248	28.3
WHIP	MACS	88803	17.0
YY1	MACS	42162	32.3
ZBTB33	spp	1934	64.8
ZEB1	spp	8304	46.5
ZNF143	MACS	81743	18.4
ZNF274	MACS	1483	1.5

Supplementary Table 1. List of DBPs from Figure 1 with their total peak counts and associated peak caller. All DBPs are from ENCODE cell line GM12878.

Supplementary Table 2

DBP	Figure 1 panel	# over-dispersed GO terms	% GO terms over-dispersed		DBP	Figure 1 panel	# over-dispersed GO terms	% GO terms over-dispersed
ATF3	a, d	630	35		PAX5	b, e	1831	100
cFOS	a, d	758	42		POL2	b, e	1828	100
ETS1	a, d	1253	68		POU2F2	b, e	1827	100
GABP	a, d	1620	88		RAD21	b, e	1755	96
JunD	a, d	692	38		RFX5	b, e	1829	100
MAX	a, d	705	39		SP1	b, e	1829	100
MEF2C	a, d	561	31		STAT3	b, e	1830	100
NF-E2	a, d	1800	98		TBP	b, e	1831	100
NFKB	a, d	1803	98		TCF12	b, e	1828	100
NRSF	a, d	493	27		USF2	b, e	1830	100
P300	a, d	1413	77		BHLHE40	c, f	1831	100
PBX3	a, d	1751	96		CHD2	c, f	1832	100
RXRA	a, d	1319	72		CTCF	c, f	1831	100
SIX5	a, d	1448	79		Ctcf	c, f	1831	100
SRF	a, d	902	49		EBF1	c, f	1832	100
TAF1	a, d	1614	88		Ezh2	c, f	1830	100
TR4	a, d	577	32		H2az	c, f	1832	100
USF1	a, d	1763	96		H3k27ac	c, f	1832	100
ZBTB33	a, d	769	42		H3k27me3	c, f	1831	100
ZEB1	a, d	1807	99		H3k36me3	c, f	1832	100
ZNF274	a, d	832	59		H3k4me1	c, f	1832	100
BATF	b, e	1821	99		H3k4me2	c, f	1832	100
BCL11A	b, e	1813	99		H3k4me3	c, f	1832	100
BCL3	b, e	1826	100		H3k9ac	c, f	1832	100
BCLAF1	b, e	1831	100		H3k9me3	c, f	1832	100
BRCA1	b, e	1824	100		MXI1	c, f	1832	100
EGR1	b, e	1825	100		PU1	c, f	1828	100
ELF1	b, e	1831	100		SMC3	c, f	1831	100
H3k79me2	b, e	1832	100		WHIP	c, f	1832	100
H4k20me1	b, e	1830	100		YY1	c, f	1832	100
MEF2A	b, e	1815	99		ZNF143	c, f	1832	100
NRF1	b, e	1829	100					

Supplementary Table 2. Significant overdispersion in peak count among genes is observed for a substantial number of GO terms in all 63 ENCODE ChIP-seq datasets from cell line GM12878. Number and percentage of GO terms (with 50-500 genes) that contain significant overdispersion in peak counts among the genes ($q \leq 0.05$). DBPs from Figure 1b,c have more peaks than DBPs from Figure 1a and thus higher power to detect significant overdispersion.

Supplementary Table 3

GO Term	# genes	total genes in term	fold enrich	p-value	q-value
nucleosome	13	58	11.64	$8.0x10^{-10}$	$2.5x10^{-7}$
protein-DNA complex	14	81	8.97	$4.3x10^{-9}$	$6.6x10^{-7}$
translation	25	314	4.10	$9.7x10^{-9}$	$1.3x10^{-5}$
DNA packaging	14	105	6.87	$1.1x10^{-7}$	$7.2x10^{-5}$
nucleosome assembly	12	74	8.35	$1.6x10^{-7}$	$7.0x10^{-5}$
chromatin assembly	12	77	8.03	$2.5x10^{-7}$	$8.0x10^{-5}$
ribosome	18	201	4.65	$3.4x10^{-7}$	$2.6x10^{-5}$
protein-DNA complex assembly	12	81	7.63	$4.2x10^{-7}$	$1.1x10^{-4}$
cellular macromolecular complex assembly	22	304	3.73	$4.6x10^{-7}$	$1x10^{-4}$
nucleosome organization	12	83	7.45	$5.4x10^{-7}$	$1x10^{-4}$

Supplementary Table 3. GO terms most strongly associated with short locus length The 500 genes with shortest locus lengths (ranging from 23bp to 5,066bp) were tested for GO term enrichment relative to all remaining genes (having a computed locus length) using DAVID Bioinformatics Resource 6.7.

Supplementary Table 4

GO Term	# genes	total genes in term	fold enrich	p-value	q-value
homophilic cell adhesion	29	130	8.65	$1.9x10^{-18}$	$3.9x10^{-15}$
nervous system development	78	1066	2.84	$2.2x10^{-17}$	$2.3x10^{-14}$
cell adhesion	60	686	3.39	$9.9x10^{-17}$	$7.6x10^{-14}$
biological adhesion	60	687	3.39	$1.0x10^{-16}$	$5.7x10^{-14}$
cell-cell adhesion	37	271	5.30	$4.3x10^{-16}$	$1.8x10^{-13}$
generation of neurons	43	549	3.04	$2.1x10^{-10}$	$4.0x10^{-8}$
calcium ion binding	58	896	2.49	$2.4x10^{-10}$	$1.4x10^{-7}$
neurogenesis	44	591	2.89	$6.1x10^{-10}$	$1.1x10^{-7}$
neuron differentiation	34	429	3.07	$1.9x10^{-8}$	$3.0\ x10^{-6}$
axonogenesis	21	191	4.26	$1.0x10^{-7}$	$1.5x10^{-5}$

Supplementary Table 4. GO terms most strongly associated with long locus length. The 500 genes with the longest locus lengths (ranging from 879 kb to 15,8 Mb) were tested for GO term enrichment relative to all remaining genes (having a computed locus length) using DAVID Bioinformatics Resource 6.7.

Supplementary Table 5

a Rank	GO term	*nearest TSS* q-value	*≤1kb from TSS* q-value	GOseq q-value
**1**	**epithelial cell differentiation**	**$1.8x10^{-6}$**	**1.0**	**$1.2x10^{-6}$**
2	adherens junction	$5.3x10^{-5}$	1.0	0.39
3	anchoring junction	$5.3x10^{-5}$	1.0	0.49
**4**	**negative regulation of sequence-specific DNA binding transcription factor activity**	**$5.5x10^{-5}$**	**1.0**	**$3.0x10^{-4}$**
**5**	**anti-apoptosis**	**$5.5x10^{-5}$**	**0.34**	**$3.2x10^{-9}$**
6	regulation of epithelial cell differentiation	$7.6x10^{-5}$	1.0	0.14
7	basolateral plasma membrane	$1.7x10^{-4}$	01.0	0.52
8	unsaturated fatty acid metabolic process	$2.9x10^{-4}$	$3.5x10^{-3}$	0.52
**9**	**icosanoid metabolic process**	**$1.8x10^{-4}$**	**$2.9x10^{-3}$**	**$4.1x10^{-4}$**
10	focal adhesion	$4.5x10^{-4}$	1.0	0.32
11	cell-substrate junction	$4.5x10^{-4}$	1.0	0.39
12	cell-substrate adherens junction	$4.5x10^{-4}$	1.0	0.35
**13**	**regulation of small GTPase mediated signal transduction**	**$8.7x10^{-4}$**	**1.0**	**$1.3x10^{-3}$**
**14**	**response to inorganic substance**	**$1.2x10^{-3}$**	**0.075**	**$4.3x10^{-4}$**
15	response to growth hormone stimulus	$1.4x10^{-3}$	1.0	1.0
**16**	**regulation of cellular component movement**	**$1.8x10^{-3}$**	**0.74**	**$5.7x10^{-6}$**
17	monocarboxylic acid metabolic process	$1.9x10^{-3}$	0.15	0.66
18	response to calcium ion	0.0024	0.33	0.14
19	regulation of anti-apoptosis	$2.9x10^{-3}$	0.76	0.63
**20**	**negative regulation of protein metabolic process**	**$3.2\ x10^{-3}$**	**0.57**	**$5.5\ x10^{-3}$**
21	response to glucocorticoid stimulus	$3.5\ x10^{-3}$	0.56	0.39
22	positive regulation of anti-apoptosis	$3.7\ x10^{-3}$	0.62	0.49
23	response to corticosteroid stimulus	$3.9\ x10^{-3}$	0.63	0.18
24	regulation of epidermal cell differentiation	4.1 x10+	1.0	0.84
25	Ras protein signal transduction	$4.1\ x10^{-3}$	1.0	0.26
26	energy reserve metabolic process	$4.2\ x10^{-3}$	1.0	0.60
**27**	**negative regulation of transcription from RNA polymerase II promoter**	**$4.4\ x10^{-3}$**	**1.0**	**$2.5x10^{-6}$**
28	actin cytoskeleton organization	$4.4\ x10^{-3}$	1.0	0.15
**29**	**vasculature development**	**$4.7\ x10^{-3}$**	**0.97**	**$7.4x10^{-16}$**
**30**	**small GTPase mediated signal transduction**	**$4.7\ x10^{-3}$**	**1.0**	**$4.9\ x10^{-3}$**

Supplementary Table 5a. Top enriched GO terms (not collapsed, with ≤500 genes) for GR ChIP-seq data (4,392 peaks) that were significantly enriched (q ≤0.05) using the *nearest TSS* locus definition. Bolded terms are significantly enriched in both ChIP-Enrich and GOseq results. The complete list of enriched GO terms for GR using the *nearest TSS* and *≤1kb from TSS* locus definition is included as a supplemental excel file, "Supplementary_table_5expanded.csv"

b Rank	GO term	*≤1kb from TSS* q-value	*nearest TSS* q-value	GOseq q-value
**1**	**negative regulation of blood coagulation**	**$3.2x10^{-7}$**	**0.077**	**0.010**
**2**	**negative regulation of coagulation**	**$4.3x10^{-7}$**	**0.15**	**0.015**
**3**	**fibrinolysis**	**$3.5x10^{-5}$**	**0.088**	**0.033**
**4**	**regulation of blood coagulation**	**$7.0x10^{-5}$**	**0.011**	**$5.4x10^{-4}$**
5	regulation of fibrinolysis	$1.4x10^{-4}$	0.073	0.11
**6**	**regulation of coagulation**	**$1.4x10^{-4}$**	**0.029**	**$9.9x10^{-4}$**
7	intrinsic to external side of plasma membrane	$1.8x10^{-4}$	0.062	0.68
8	leukotriene metabolic process	$2.2x10^{-4}$	$6.3x10^{-3}$	1.0
**9**	**positive regulation of coagulation**	**$3.1x10^{-3}$**	**0.061**	**0.028**
10	anchored to plasma membrane	$2.1x10^{-3}$	0.39	1.0
**11**	**regulation of wound healing**	**$2.9x10^{-3}$**	**$6.4x10^{-3}$**	**$1.4X10^{-4}$**
**12**	**regulation of response to external stimulus**	**$2.9x10^{-3}$**	**0.014**	**$5.0X10^{-5}$**
**13**	**positive regulation of coagulation**	**$3.0x10^{-3}$**	**0.061**	**0.028**
**14**	**positive regulation of leukocyte chemotaxis**	**$3.5\ x10^{-3}$**	**0.092**	**0.017**
15	platelet alpha granule lumen	$4.7\ x10^{-3}$	0.25	0.61
16	secretory granule lumen	$4.7\ x10^{-3}$	0.29	0.63
17	cytoplasmic membrane-bounded vesicle lumen	$5.1\ x10^{-3}$	0.25	0.64
18	ameboidal cell migration	$5.2\ x10^{-3}$	0.31	0.94
19	regulation of nuclease activity	$5.2\ x10^{-3}$	0.083	0.66
**20**	**cellular response to biotic stimulus**	**$5.2x10^{-3}$**	**$6.1x10^{-3}$**	**$3.7x10^{-3}$**
21	vesicle lumen	$5.5\ x10^{-3}$	0.20	0.68
22	nucleotide-binding domain, leucine rich repeat containing receptor signaling pathway	$6.1\ x10^{-3}$	0.15	0.32
23	peptidyl-glutamic acid carboxylation	$6.9\ x10^{-3}$	0.11	1.0
**24**	**regulation of leukocyte chemotaxis**	**0.011**	**0.18**	**0.028**
25	long-chain fatty acid transport	0.011	0.028	0.12
**26**	**second-messenger-mediated signaling**	**0.012**	**0.66**	**0.047**
**27**	**positive regulation of leukocyte migration**	**0.014**	**0.20**	**0.031**
28	carboxylic acid transport	0.014	0.18	0.39
29	external side of plasma membrane	0.015	0.63	1.0
30	endoplasmic reticulum unfolded protein response	0.069	0.015	0.29

Supplementary Table 5b. Top enriched GO terms (not collapsed, with ≤500 genes) for GR ChIP-seq data (4,392 peaks) that were significantly enriched (q ≤0.05) using the *≤1kb from TSS* locus definition. Bolded terms are significantly enriched in both ChIP-Enrich and GOseq results. The complete list of enriched GO terms for GR using the *nearest TSS* and *≤1kb from TSS* locus definition is included as a supplemental excel file, "Supplementary_table_5expanded.csv"

Supplementary Results: Figures

Supplementary Figure 1

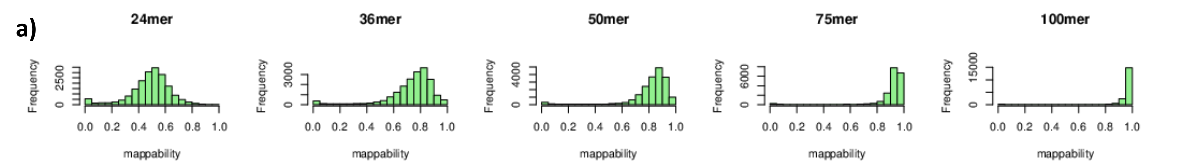

**b)** GO Terms whose Genes' Loci Have Higher Mappability			
GO Term	# Genes	P-value	Q-value
Organ morphogenesis	642	2.6E-22	5.5E-19
Central nervous system development	454	2.9E-19	3.0E-16
Neurogenesis	634	1.4E-18	9.8E-16
Neuron differentiation	534	2.7E-18	1.4E-15
Cell development	786	5.5E-18	2.3E-15
Generation of neurons	589	1.6E-17	5.6E-15
Skeletal system development	272	2.6E-16	7.8E-14
Regionalization	217	1.9E-15	4.9E-13

**c)** GO Terms whose Genes' Loci Have Lower Mappability			
GO Term	# Genes	P-value	Q-value
Olfactory receptor activity	114	1.6E-11	7.0E-09
Sensory perception of smell	131	1.3E-09	6.3E-08
Cellular defense response	60	3.0E-08	9.0E-07
Sensory perception of chemical stimulus	167	3.7E-08	1.1E-06
Oxygen binding	44	7.7E-08	8.7E-06
Cellular response to xenobiotic stimulus	35	2.2E-07	5.1E-06
Xenobiotic metabolic process	35	2.2E-07	5.1E-06
Electron carrier activity	156	4.9E-07	3.0E-05

Supplementary Figure 1. Gene loci with high (or low) average mappability are enriched for specific Gene Ontology terms. (a) Distribution of human (hg19) mappability scores (calculated as the average mappability for each gene locus using the *nearest TSS* locus definition) for five different sequencing read lengths. (b) Most significantly enriched GO terms associated with high mappability using 50mer reads (c) Most significantly enriched GO terms associated with low mappability using 50mer reads. GO biological processes and molecular functions were tested using the LRpath gene set enrichment program [7].

Supplementary Figure 2

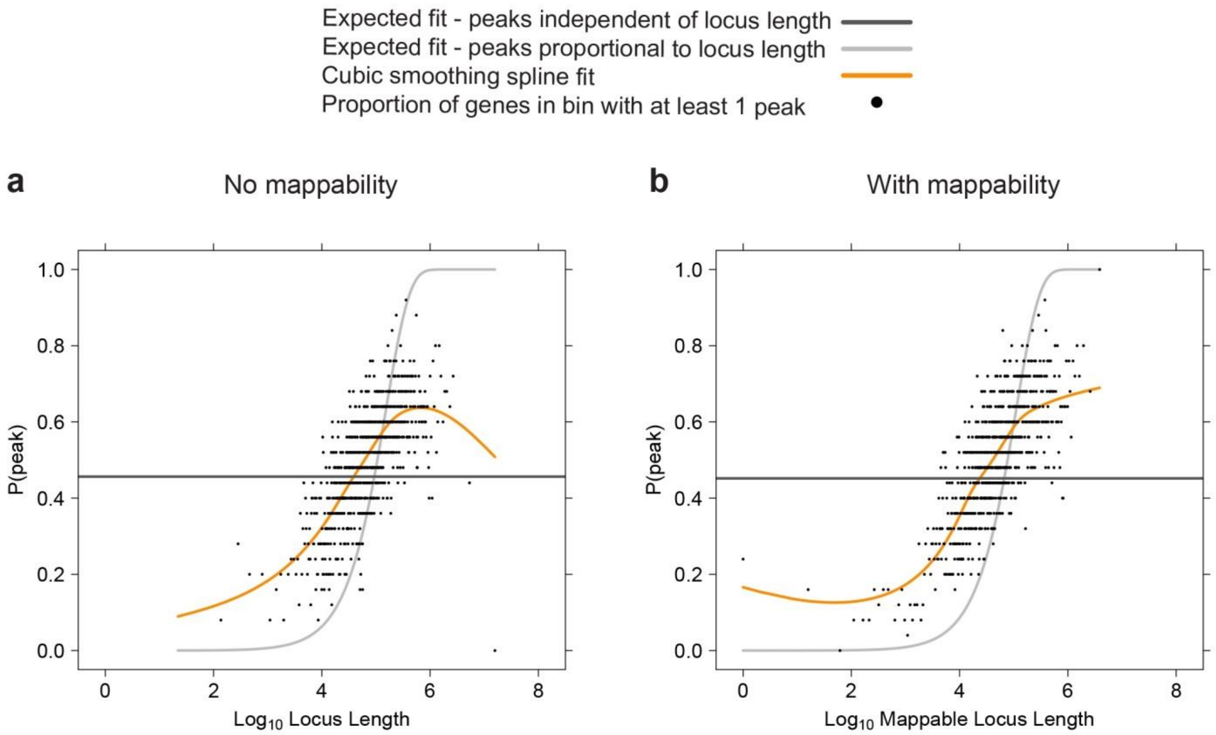

Supplementary Figure 2. Using the mappable locus length (locus length x mappability) tends to improve the fit of the binomial cubic smoothing spline in the model, illustrated here with PAX5. Adjusting for mappability often shifts up the spline fit for the longest locus lengths; the dip in the fit without mappability is due to outlier points with long locus length and very low mappability.

Supplementary Figure 3

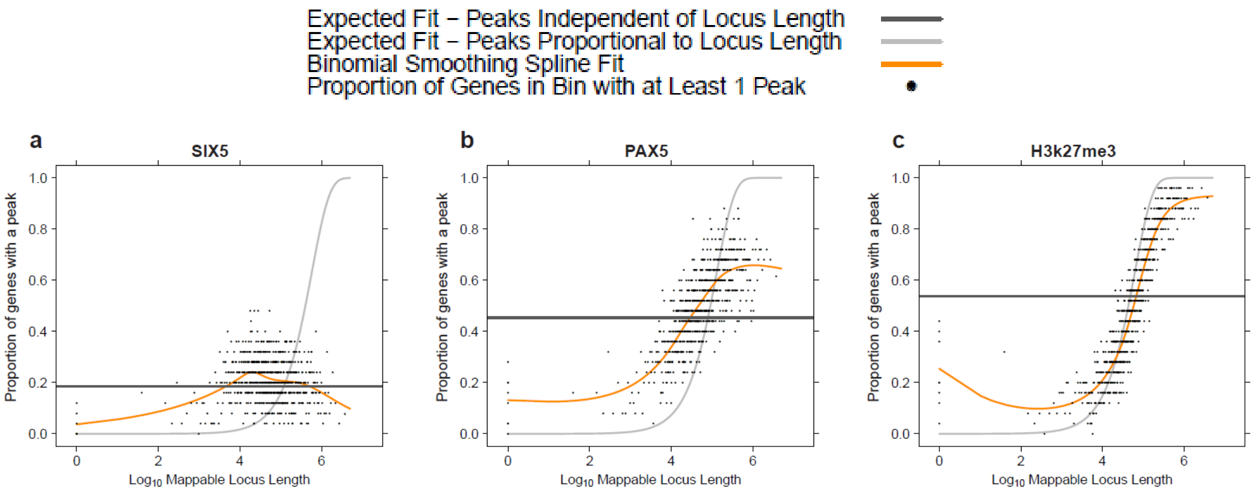

Supplementary Figure 3. SIX5, PAX5, and H3K27me3 have different gene locus-length-to-peak presence relationships. SIX5 (also in Figure 1a) has a weak relationship; PAX5 (also in Figure 1b) has a mid-level relationship; H3K27me3 (also in Figure 1c) has a strong relationship. The relationships between locus length and proportion of genes with a peak were estimated using a binomial cubic smoothing spline (orange line). Expected line if no relationship between presence of ≥ 1 peak and log10 locus length (dark gray, satisfies Fisher's exact test assumptions). Expected line if number of peaks observed is proportional to locus length (light gray, binomial test assumption). For visualization only, each point is the proportion of genes assigned a peak within sequential bins of 25 genes.

Supplementary Figure 4

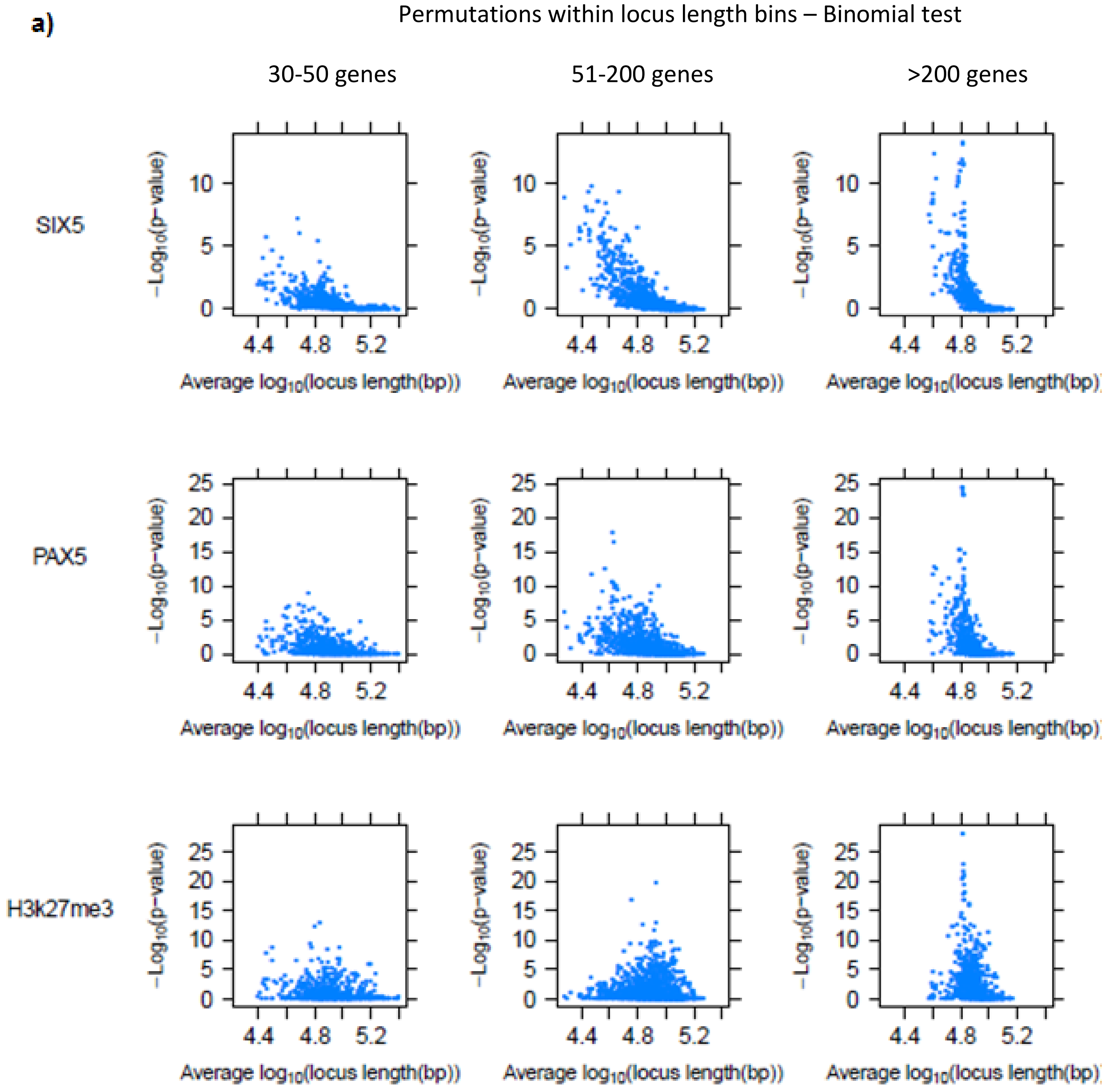

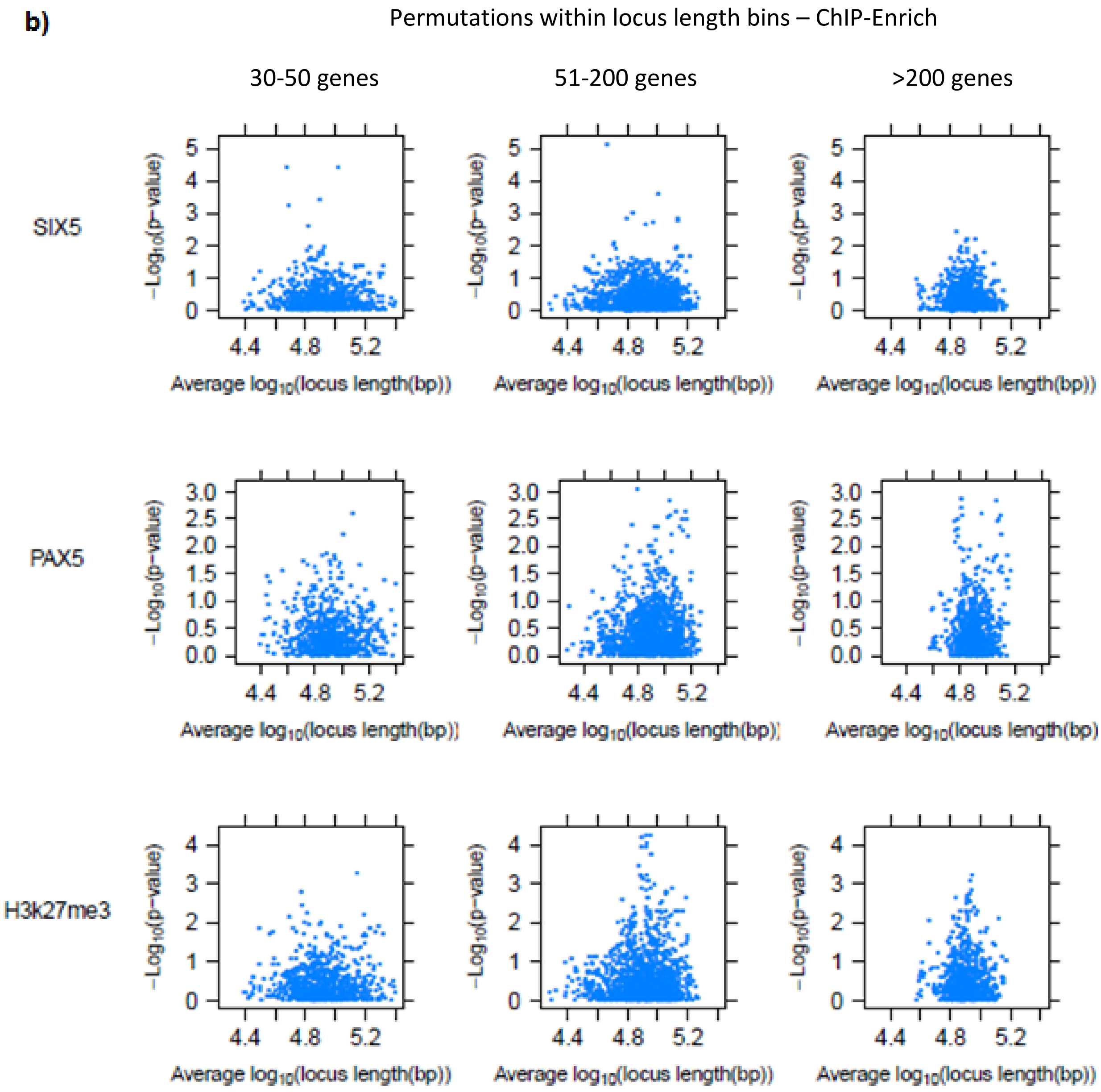

Supplementary Figure 4. The binomial test tends to identify gene sets with short locus length as significant ($p < 0.05$), especially for SIX5. Panel **(a)** shows the $-\log_{10}$ p-values from the binomial test versus the average $\log_{10}$ locus length of each gene set tested. Each row shows results from a permutation of the DBP dataset, where the original DBP dataset has been permuted by shuffling genes within bins of locus length. Each column subdivides all gene sets by their number of genes: the first column has gene sets with 30-50 genes, the next 51-200 genes, and the last > 200 genes. Panel **(b)** shows plots as in (a) for ChIP-Enrich. The binomial test shows a trend of much larger $-\log_{10}$ p-values for gene sets with low average $\log_{10}$ locus length, and this trend is most pronounced for sets of genes with fewer than 200 genes (first and second columns.) ChIP-Enrich does not show this trend for any of the three datasets tested.

Supplementary Figure 5

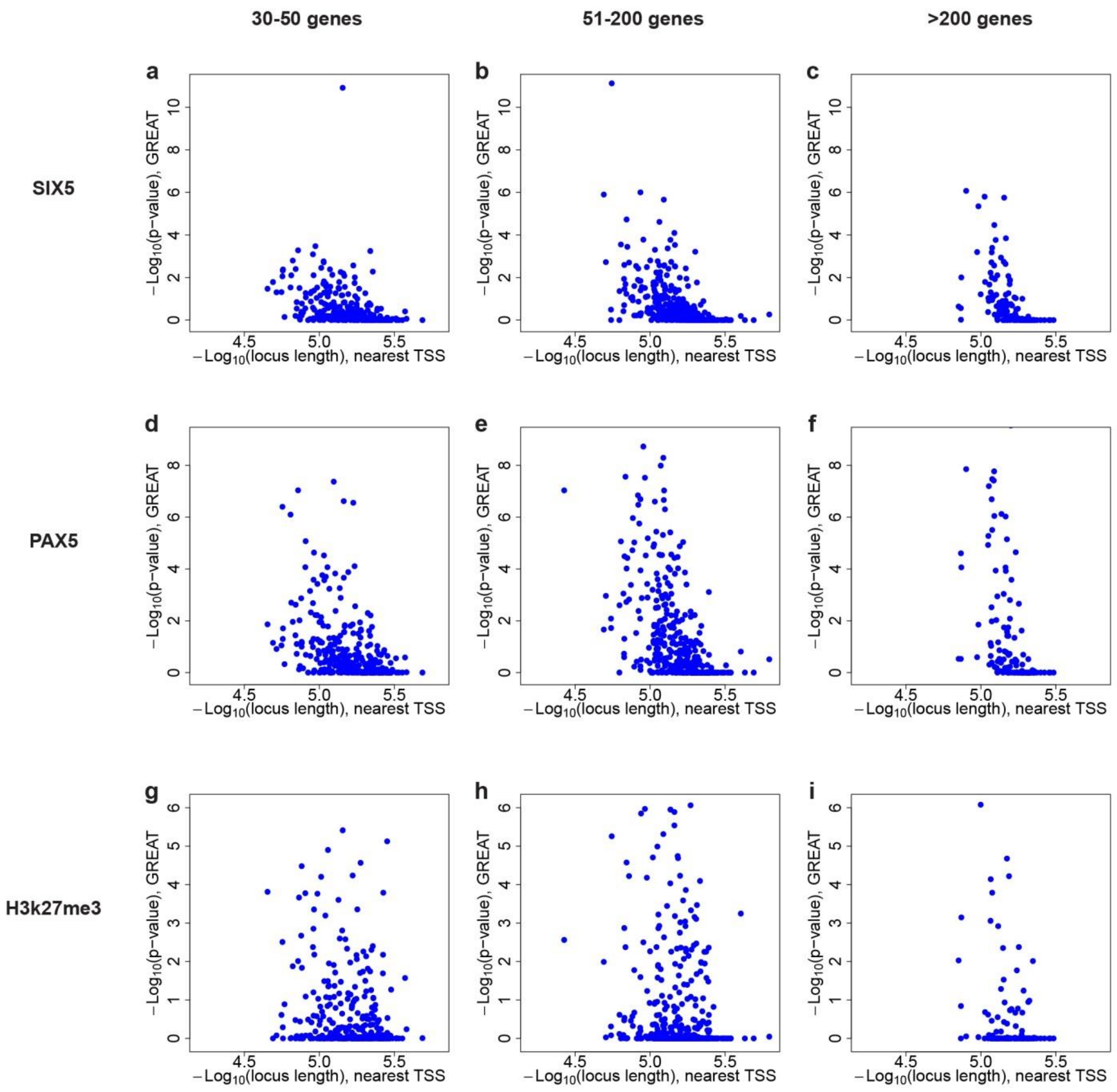

Supplementary Figure 5. **The GREAT website test tends to detect gene sets with shorter than average locus length, especially for SIX5.** Plots show the $–\log_{10}$ p-values from the GREAT test versus the average $\log_{10}$ locus length of each gene set tested. Each row show results from a permutation of the DBP dataset, where the original DBP dataset has been permuted by shuffling genes within bins of locus length. Each column subdivides all gene sets by their number of genes. Gene set enrichment testing using GREAT on each set of permuted peaks from SIX5, PAX5, and H3k27me3 found significantly enriched GO terms (FDR≤0.05), when none should have been detected**.** The trend with locus length was again greatest for SIX5 and least for H3k27me3.

Supplementary Figure 6

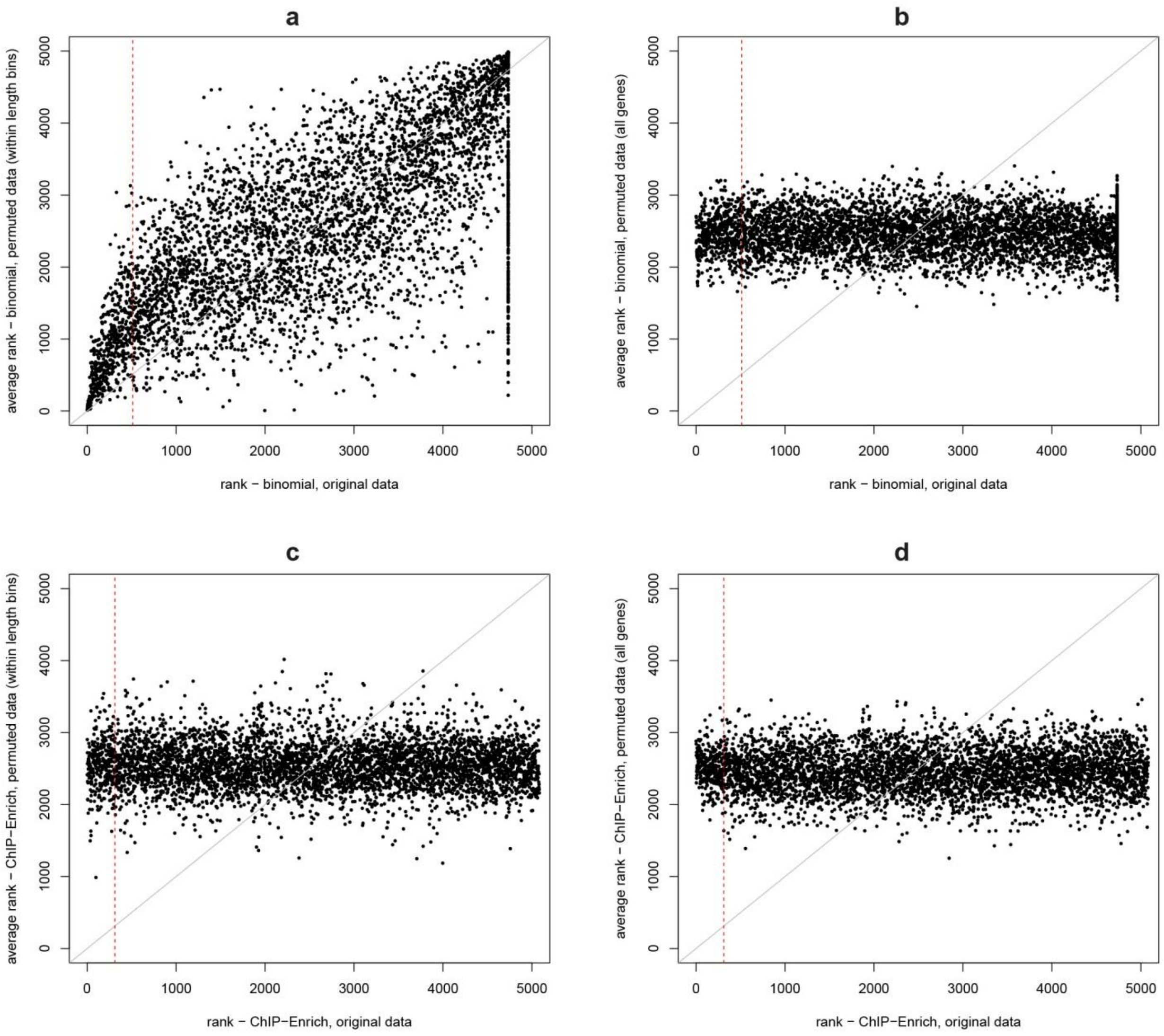

Supplementary Figure 6. **For SIX5 the ranks of the binomial test results from the original data are highly correlated the average ranks from 25 permutations (within locus length bins) of the original data (Spearman r = 0.71). For ChIP-Enrich the rank of test results from original data and the average rank permuted data are not correlated.** The fact that this correlation is not observed in (b, permutations across all genes), implies that the correlation is due to the locus length bias. Each plot compares the average ranks of results from 25 permutations to rank in the original data, and the red dash line indicates the highest rank where FDR≤0.05 for the original data. (a) Binomial test results for permutations within locus length bins. Of the 509 significantly enriched (FDR≤0.05) GO terms (with ≤500 genes) using the original, non-permuted data, 413 (81.1%) were also significantly enriched in at least one of the permuted data sets. Of the 4,325 not significantly enriched GO terms, only 583 (13.5%) were enriched in at least one of the permuted data sets. (b) Binomial test results from permutations across all genes. The average ranks of the binomial test results are not correlated with the ranks of the original data (Spearman r=-0.06), indicating that the correlation in (a) is due to the confounding by locus length. ChIP-Enrich test results from (c) permutations within locus length bins and (d) permutations across all genes, respectively. In both permutation scenarios, the ranks of ChIP-Enrich results from permuted and the original data were not correlated (Spearman r=-0.02 and -0.005, respectively).

Supplementary Figure 7

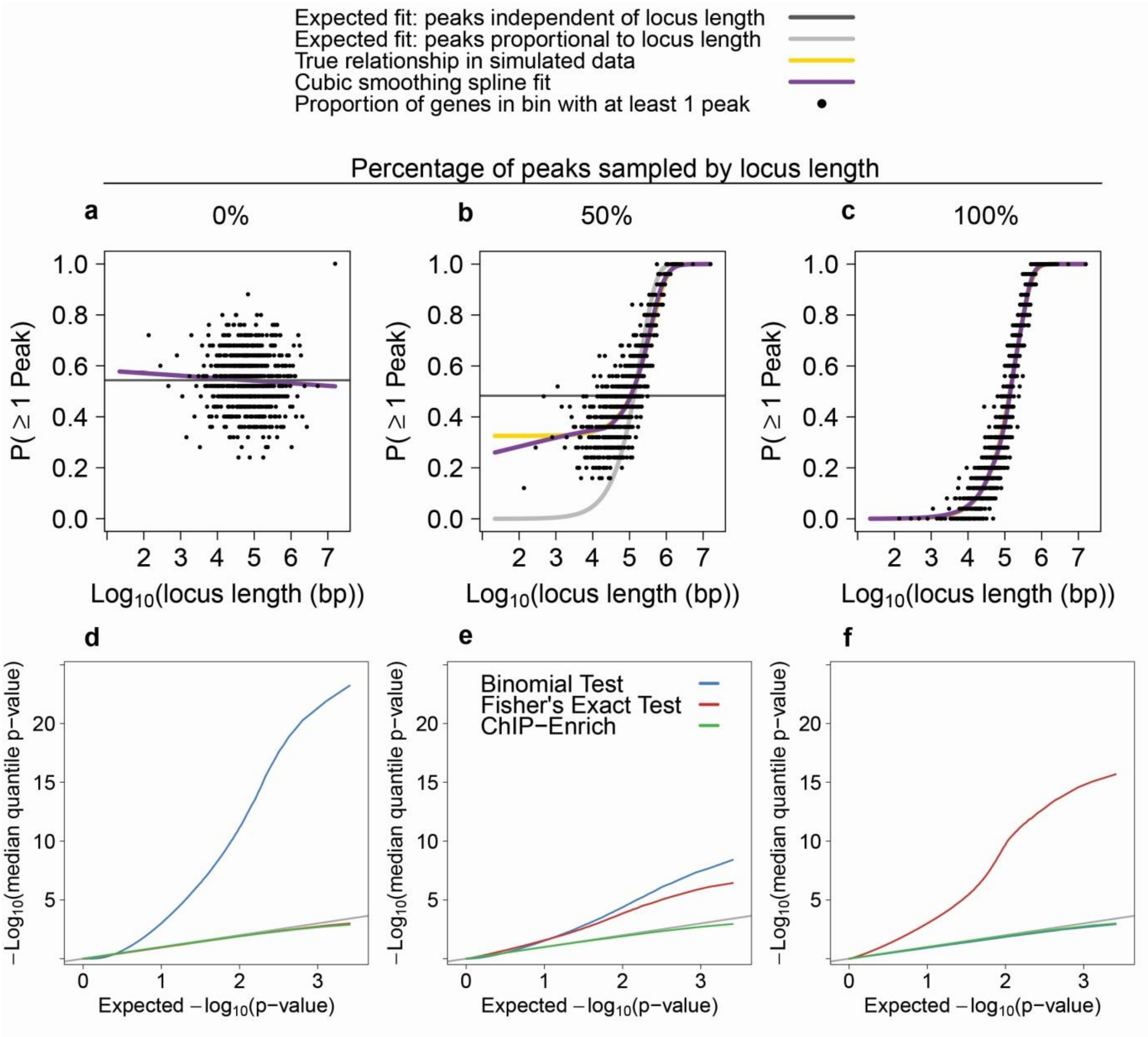

Supplementary Figure 7. Relationship in simulated datasets between locus length and presence of at least one peak (a-c), and QQ-plots showing the type 1 error rate of Fisher's exact test, the binomial test, and ChIP-Enrich under these relationships (d-f). Simulated datasets of 10,000 peaks and 0% (a, d), 50% (b, e), or 100% (c, f) of peaks sampled in proportion to locus length. **Top row (a-c) -** For visualization, each point is a bin of 25 genes, plotted as the average proportion of genes having a peak within the bin against the average $\log_{10}$ locus length. The dark grey horizontal line represents the model where peaks occur within genes with no relationship to their locus length. The light grey line represents the probability of a locus having ≥1 peak if peaks are randomly distributed across the genome (binomial test assumption). The purple line is a binomial smoothing spline fit to the underlying data (the 0/1 vector denoting whether a peak was assigned to a gene vs. the $\log_{10}$ locus length of each gene). The yellow line represents the known relationship that exists in the simulated data. **Bottom row (d-f)** – QQ plots showing Fisher's exact and the binomial test represent two extreme assumptions for enrichment testing for ChIP-seq data, while ChIP-Enrich empirically estimates the correct balance between these two extremes. Incorrect assumptions at either end leads to biased significance levels. Median p-values (solid lines) are shown for 1000 simulations of Fisher's exact test, ChIP-Enrich, and the binomial test.

Supplementary Figure 8

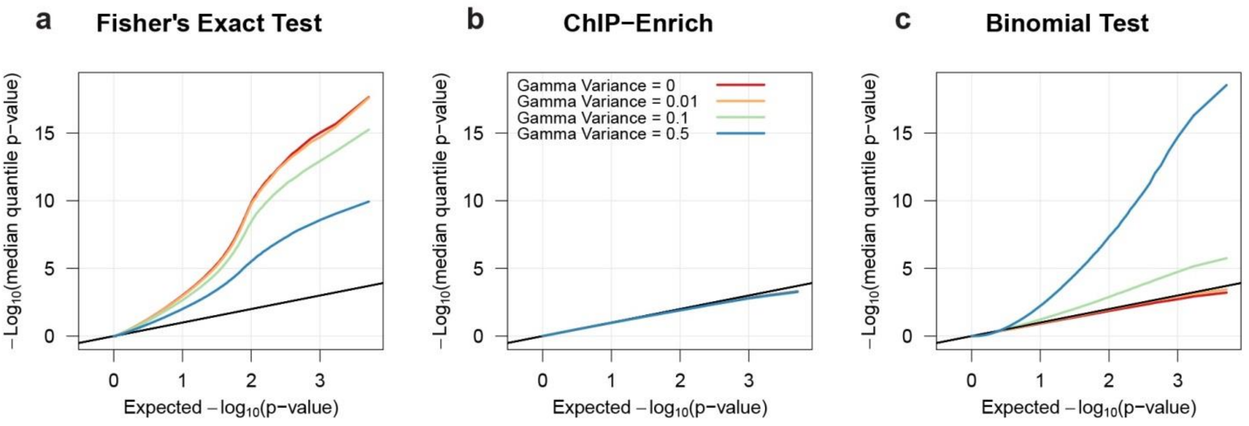

Supplementary Figure 8. Increasing overdispersion in peak counts among genes increases the type 1 error rate of the binomial test, decreases type 1 error for Fisher's exact test, and has no effect on ChIP-Enrich. QQ plots of expected versus observed $-\log_{10}$(p-values) for (a) Fisher's exact test, (b) ChIP-Enrich, and (c) the binomial test. Increasing levels of overdispersion, modeled using a gamma distribution, were assessed ranging from no overdispersion (red) to a gamma distribution with variance = 0.5 (blue). Red line (no overdispersion) represents the same simulation as that in Supplementary Figure 7c,f. Simulated datasets of 10,000 peaks were used, and median p-values for 1000 simulations are shown.

Supplementary Figure 9

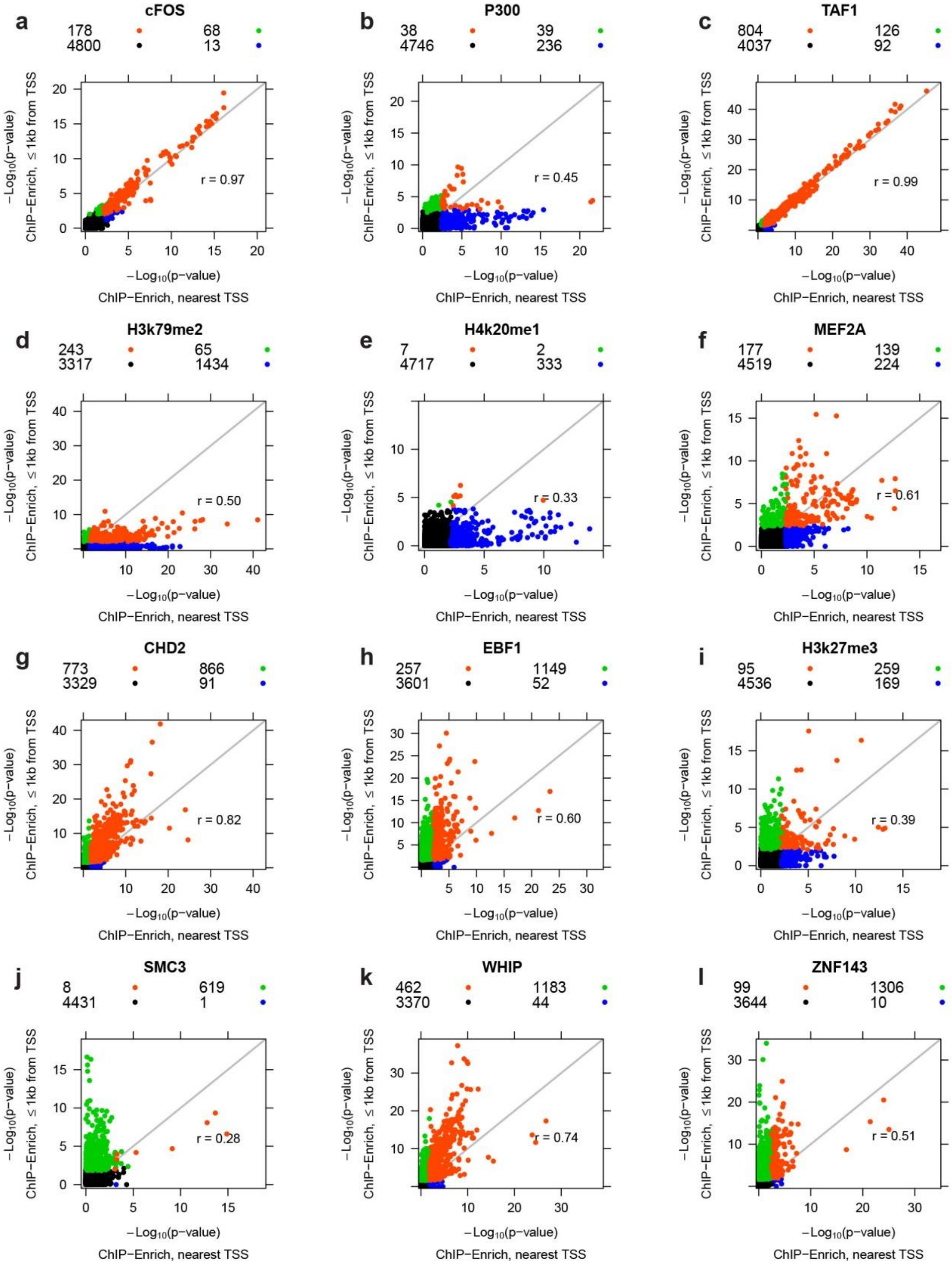

Supplementary Figure 9. Gene set enrichment testing using ≤ *1kb from TSS* and *nearest TSS* locus definitions often identifies very different sets of significant GO terms for the same DBP. Comparison of –$\log_{10}$(p-values) from testing GO terms with ChIP-Enrich using ≤ *1kb from TSS* versus *nearest TSS* locus definitions in ENCODE data for the GM12878 cell line. GO terms with: FDR ≤.05 for ≤ *1kb from TSS* only (green); FDR ≤.05 for *nearest TSS* only (blue); FDR ≤.05 for ≤ *1kb from TSS* and *nearest TSS* (orange); FDR >.05 in both analyses (black). r: Pearson correlation coefficient. DBPs are arranged by groupings in Figure 1A, (a-c) are DBPs with low number of peaks, (d-f) are DBPs with medium number of peaks, and (g-l) are DBPs with high number of peaks. The numbers associated with each colour in the legends indicate the number of each coloured dot in each figure.

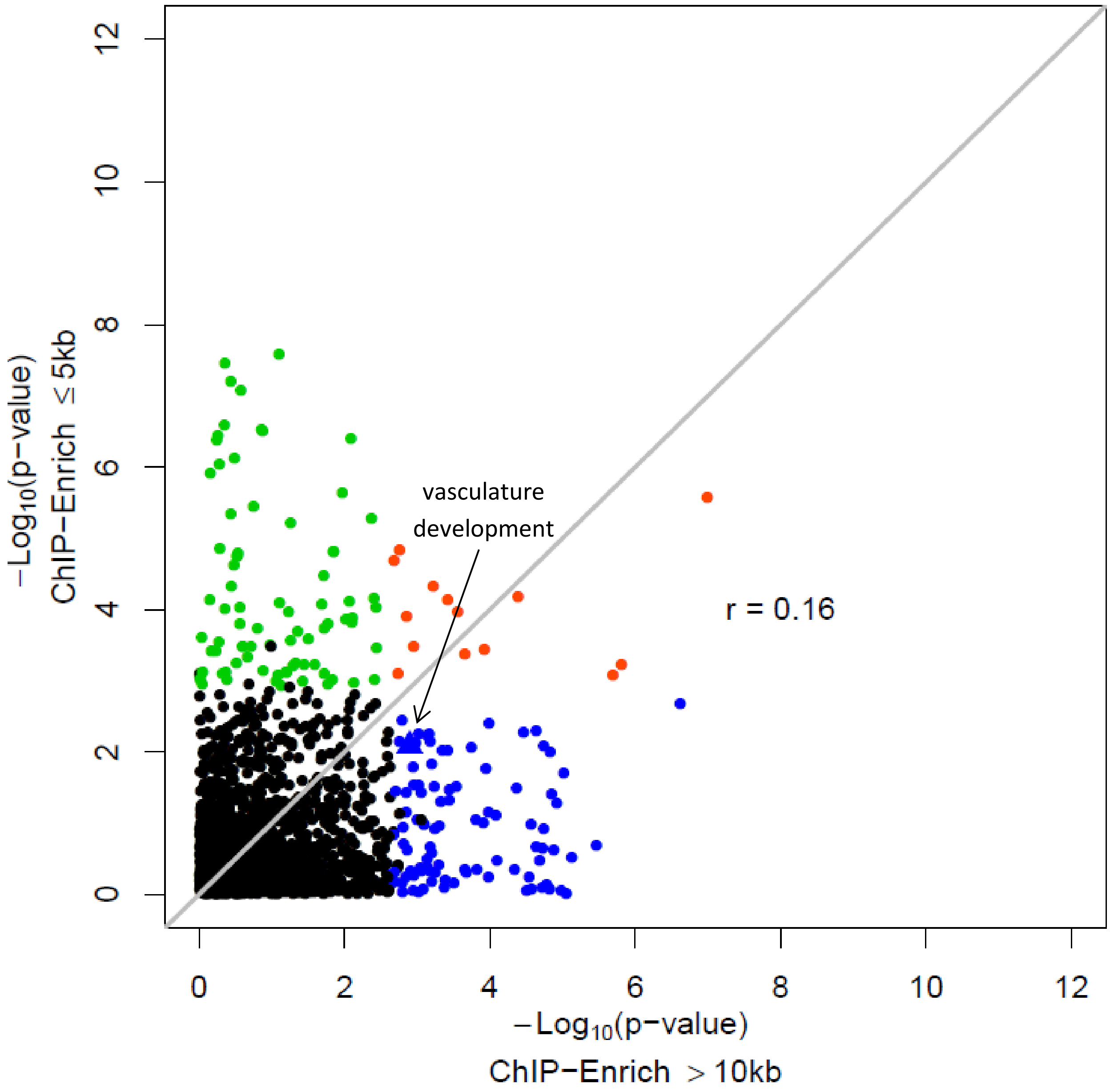

Supplementary Figure 10. A comparison of ChIP-Enrich GO term enrichment results for GR using peaks ≤5kb from the TSS and peaks >10kb from the TSS. Only 14 gene sets were significantly enriched (q≤0.05) in both tests. Vasculature development (shown as the blue triangle) was only significant using peaks *>10kb from the TSS*. GO terms with: FDR ≤.05 for *≤ 5kb from TSS* only (green); FDR ≤.05 for *>10kb from TSS* only (blue); FDR ≤.05 for *≤ 5kb from TSS* and *≥10kb from TSS* (orange); FDR >.05 in both analyses (black). r: Pearson correlation coefficient.
